# Recent Progress of Photodetectors and Optoelectronic Synapses Based on Metal Oxide Thin-Film Transistors

**DOI:** 10.3390/ma19173626

**Published:** 2026-08-26

**Authors:** Junyan Ren, Lingyan Liang, Hongtao Cao

**Affiliations:** Laboratory of Atomic-Scale and Micro & Nano Manufacturing, Ningbo Institute of Materials Technology and Engineering (NIMTE), Chinese Academy of Sciences (CAS), Ningbo 315201, China

**Keywords:** metal oxide thin-film transistor, photodetector, optoelectronic synapse, persistent photoconductivity

## Abstract

Metal oxide thin-film transistors (MO TFTs) have drawn wide interest in photodetectors and optoelectronic synaptic devices owing to their wide bandgap, low off-state current, high optical transparency, low-temperature processing, and large-area uniformity. Gate modulation in the TFT structure can tune the channel’s initial state and interfacial electric field, enhancing the tunability of photogenerated carrier transport, defect trapping/release, and interfacial charge regulation. This article reviews the progress of MO TFT photodetectors and optoelectronic synaptic devices, and examines the roles of light absorption, carrier transport, defect-related carrier dynamics, interfacial charge control, and persistent photoconductivity in different device functions. For photodetectors, key goals include broadening the response spectrum, reducing dark current, improving spectral selectivity, and enhancing response stability. For optoelectronic synaptic devices, post-illumination conductance retention and slow relaxation enable memory retention and synaptic weight modulation. Thus, rather than being separate, photodetection and optoelectronic synapses are functional extensions of the MO TFT optoelectronic response under different application targets. This article further discusses the synergy between these two functions in array sensing, visual preprocessing, and intelligent vision systems. Future development requires advances in targeted defect engineering, interface and structure optimization, array uniformity, standardized evaluation, and device–circuit–algorithm co-design for low-power, integrable intelligent vision hardware.

## 1. Introduction

**Figure 1:**
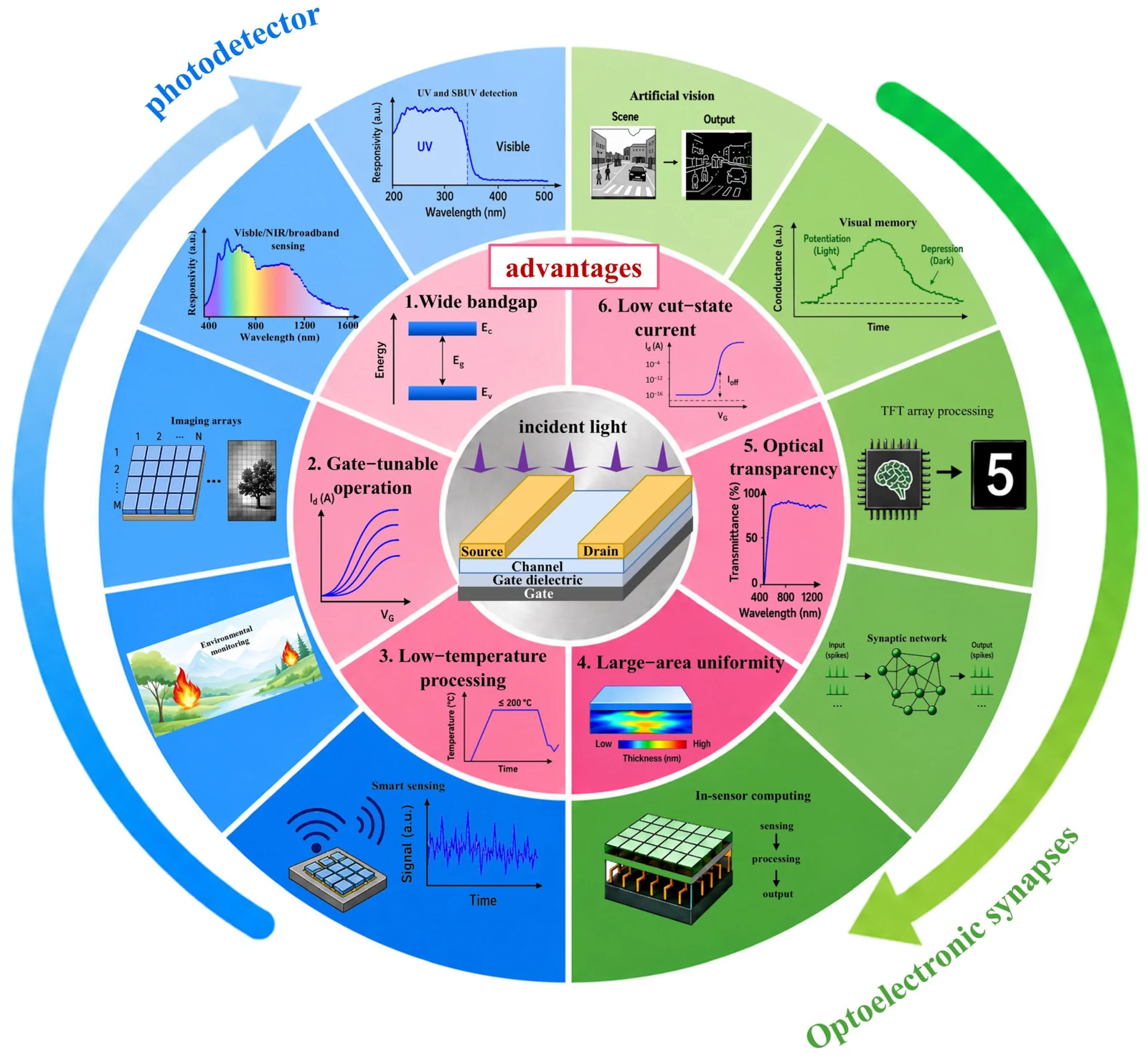
Advantages of metal oxide thin-film transistors and their applications in photodetection and optoelectronic synapses.

Metal oxide thin-film transistors (MO TFTs) have attracted widespread attention in the field of display backplanes [1,2,3]. Metal oxide semiconductors, represented by amorphous indium–gallium–zinc oxide (a-IGZO), feature high electron mobility, low off-current, good optical transparency, compatibility with low-temperature fabrication, and the ability to deposit uniformly over large areas. These properties enable them to meet the demands of active-matrix displays for high resolution, low power consumption, and large-area fabrication. As display technology evolves toward high refresh rates, flexibility, transparency, and multifunctional integration, MO TFTs are no longer limited to serving as pixel switches or driver devices. Their sensitivity to light, electricity, and interface states has also led to their gradual expansion into fields such as photodetection, visual sensing, and neuromorphic devices (Figure 1).

Compared to traditional two-terminal optoelectronic devices, the TFT structure offers an additional gate control degree of freedom, allowing the initial channel state, dark current, responsivity and recovery process to be modulated by an external electric field [4]. Consequently, MO TFTs can serve not only as photodetectors (PDs) but also provide a device foundation for Optoelectronic Synapse (OES) via optical writing, electrical reading, and state modulation.

The photoelectric behavior of MO TFTs is simultaneously influenced by band structure and defect-related carrier dynamics. The wide bandgap offers advantages in UV and solar-blind ultraviolet (SBUV) photodetection but also limits visible and near-infrared response. Defect states and interface states alter the trapping, release, and recombination processes of photogenerated carriers, thereby affecting the responsivity, response speed, and conductance retention of the device. For PDs, persistent photoconductivity (PPC) typically implies response lag and signal residue; for OESs, moderate conductance retention can serve as the basis for simulating memory and plasticity. Therefore, regulating light absorption, carrier transport, and charge relaxation processes is key to understanding and optimizing MO TFT optoelectronic devices.

Currently, significant progress has been made in both MO TFT PDs and OESs. The former primarily focuses on extending the response band, improving responsivity, suppressing dark current, optimizing response speed, and achieving spectral selectivity. The latter pays more attention to conductance modulation induced by optical stimulation, synaptic plasticity, state retention, erasability, and low-power operation. Although their evaluation metrics and application goals differ, their performance evolution is closely related to light absorption, carrier transport, defect trapping, interface charge modulation, and gate control. Therefore, based on separately reviewing the research progress in PDs and OESs, it is necessary to further analyze the connections, differences, and synergistic development trends between the two types of functions from the perspective of common physical processes and device platforms. Such a review helps to more clearly understand the role of MO TFTs in optical signal detection, state retention, and intelligent visual preprocessing, and also provides a reference for subsequent device structure design and system integration.

The scope of this paper is primarily limited to TFT optoelectronic devices that use MO semiconductors as the channel or key optoelectronic control layer, with a focus on IGZO, ZnO, In_2_O_3_, SnO_2_, Ga_2_O_3_, and their multi-component oxide systems. The literature review primarily covers relevant research from the past decade or so, with an emphasis on representative advances in areas such as MO TFT PDs, OES devices, PPC, and smart vision arrays. Relevant literature was primarily retrieved through Web of Science, Google Scholar, and journal websites.

Previous reviews have focused on transparent MO TFTs [5], PPC in MO [6], photonic artificial synapses [7], and neuromorphic visual devices [8], among other areas. These studies have provided an important foundation for understanding the characteristics of oxide semiconductor materials, the PPC mechanism, and the development of photonic synaptic devices. Compared to existing reviews, this paper focuses on MO TFTs as a common device platform, placing photodetectors and photonic synaptic devices within the same photonic physics framework for discussion. The focus of this paper is not merely on the individual performance advancements of these two device types, but rather on further analyzing how processes such as light absorption, carrier transport, defect trapping and release, interfacial charge regulation, and gate voltage modulation exhibit different functional significance under different application objectives. In particular, PPC typically causes response hysteresis and signal retention in PDs, whereas in OES devices it can be utilized for memory retention and weight modulation; this functional difference serves as a key link connecting the two device classes.

Based on the above background, this review discusses the recent research progress of MO TFT PDs and OESs. It first introduces the fundamental photoelectric physical processes in MO TFTs, providing a basis for subsequent device performance analysis. Then, it summarizes the research progress in MO TFT PDs and OESs, focusing on their performance metrics, realization mechanisms, optimization strategies, and main challenges. On this basis, it further analyzes the synergistic integration of photodetection and optoelectronic synaptic functions at the single-device, array, and system levels. Finally, it provides an outlook on the development directions of this field for intelligent vision applications.

## 2. Fundamental Photoelectric Physical Processes in MO TFTs

The photoresponse of MO TFTs originates from changes in channel conductance upon illumination. Unlike simple two-terminal PDs, the gate electrode in the TFT structure can modulate the initial channel state and carrier transport processes, thereby altering the current response and its relaxation behavior before and after illumination. Therefore, MO TFTs can function not only as PDs converting optical signals into electrical output but also as OES devices utilizing the residual conductance and slow decay after illumination to simulate memory and plasticity.

Understanding the photoelectric physical processes in MO TFTs requires simultaneous attention to two aspects. On one hand, the material bandgap, defect states, and interface states determine whether light is effectively absorbed and whether photogenerated carriers can participate in channel transport. On the other hand, the gate control characteristics of the TFTs determine the electrical state under which the photoresponse is read, further influencing dark current, photocurrent, response speed, and conductance retention. This chapter first outlines the basic photoresponse processes in MO TFTs, then discusses the influence of gate modulation on the photoresponse, and finally focuses on analyzing the formation mechanism of PPC and its different roles in PD and OES applications.

### 2.1. Basic Photoresponse Processes

An MO TFT typically consists of a gate electrode, gate dielectric, metal oxide semiconductor channel, and source/drain electrodes. Under dark conditions, the channel carrier concentration is primarily determined by material intrinsic defects, interface charges, and the applied gate voltage. When light illuminates the channel or related functional layers, the material absorbs the incident light and generates photogenerated electrons and holes. For n-type MO TFTs, photogenerated electrons can participate in channel transport, increasing the source-drain current; photogenerated holes may be trapped by internal defect states, interface states, or surface adsorbates in the channel, thereby altering the local potential distribution and channel carrier concentration.

**Figure 2:**
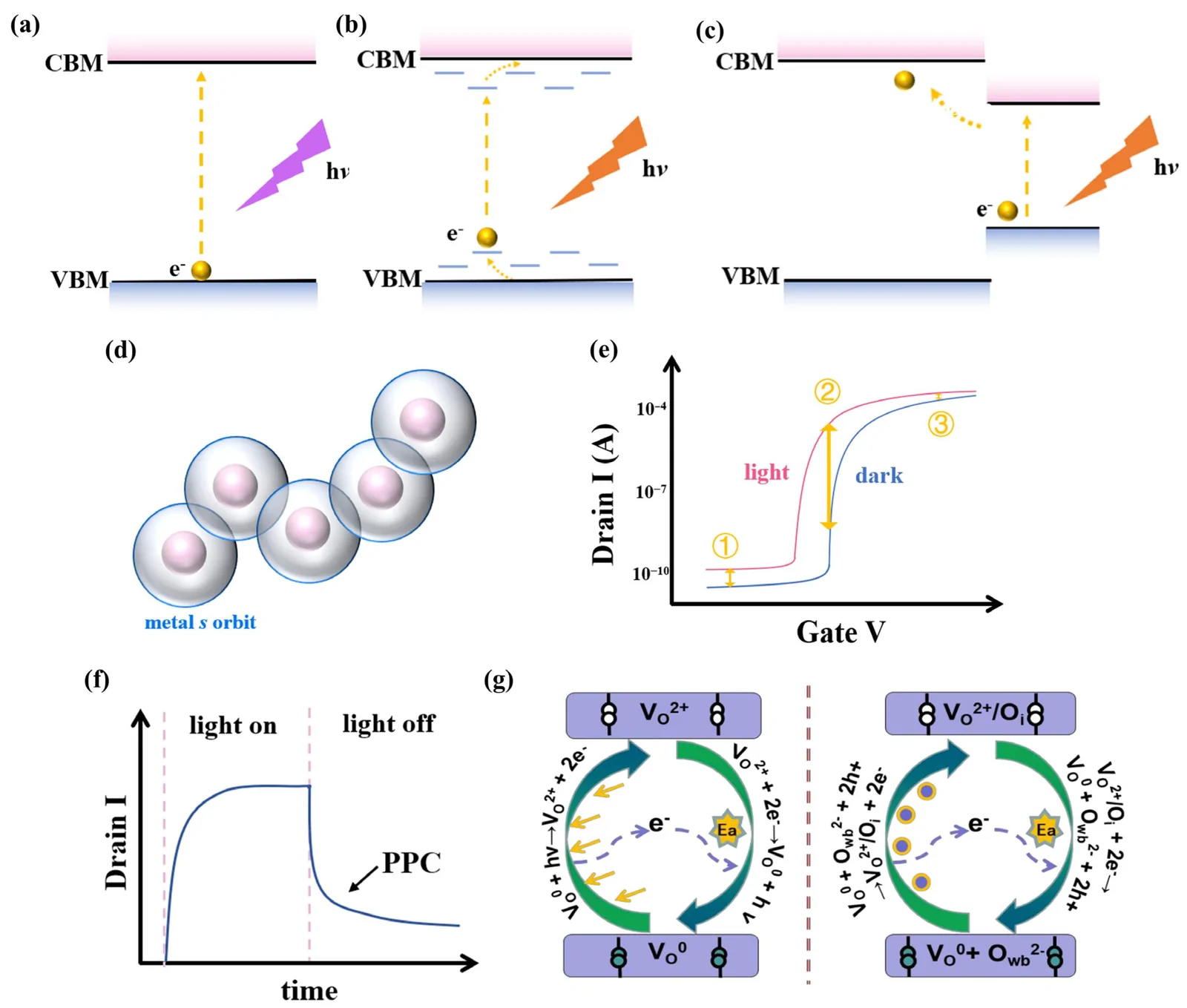
(**a**) Intrinsic light absorption, (**b**) defect-assisted light absorption and (**c**) absorption in composite photosensitive layers in metal oxides. (**d**) Metal s-orbital connectivity in amorphous disordered structures. (**e**) Light and dark currents at different gate voltages (1. off-state, 2. subthreshold region, 3. on-state). (**f**) I-T curves for devices with PPC. (**g**) Dynamic dissociation and recovery mechanism of oxygen vacancy [9]. Reproduced with permission from Ref. [9]. Copyright © 2020 John Wiley and Sons.

Light absorption is the starting point of the photoresponse. When the incident photon energy exceeds the material bandgap, valence band electrons can be excited to the conduction band, forming electrons and holes (Figure 2a). This process is the main source of intrinsic photoresponse in wide-bandgap MOs in the UV region. MOs such as ZnO, IGZO, and Ga_2_O_3_ typically have relatively wide bandgaps, so their intrinsic photoresponse is mostly concentrated in the UV, deep UV (DUV), or SBUV regions. For such materials, a wider bandgap is beneficial for suppressing visible light background interference but limits the device’s direct absorption capability in the visible and near-infrared (NIR) regions.

When the incident photon energy is lower than the material bandgap, a single wide-bandgap oxide usually struggles to produce significant intrinsic absorption. In this case, visible or NIR response often relies on additional absorption paths. For example, oxygen vacancies, band tail states, deep-level defects, or impurity states can introduce intermediate energy levels within the bandgap, allowing low-energy photons to generate photocarriers through sub-bandgap transitions (Figure 2b). Narrow-bandgap oxides; heterojunctions; and composites with photosensitive layers such as quantum dots, organic semiconductors, perovskites, or two-dimensional materials can also extend the device’s visible or broadband spectral response (Figure 2c).

After photogenerated carriers are generated in an MO TFT, whether they can be converted into effective output current depends on their separation, transport, and collection processes. The source-drain electric field drives carrier transport along the channel direction. Channel mobility, carrier concentration, and defect scattering collectively influence the magnitude of the photocurrent. Since many MO TFT channels utilize amorphous or low-crystalline films to meet the requirements of low-temperature, large-area, and uniform preparation, the carrier transport characteristics in the amorphous state are particularly important. The conduction band of MOs is mainly contributed by the s orbitals of metal cations, and electron transport is relatively less sensitive to local structural disorder, allowing amorphous MOs to still achieve good electron mobility [10] (Figure 2d). Higher mobility facilitates the rapid transport and collection of photogenerated electrons, but excessively high free-carrier concentration can also increase dark current and reduce the photo-to-dark current ratio.

### 2.2. Influence of Gate Voltage on Photoresponse and Charge Relaxation Processes

The key feature distinguishing TFT structures from two-terminal PDs is the gate’s ability to modulate the channel state. The gate voltage can alter the initial carrier concentration, the channel potential barrier, and the interface electric field distribution, thereby affecting the source-drain current change before and after illumination. Therefore, the photoresponse in MO TFTs depends not only on the absorption capability of the material itself but also on the electrical operating state of the device.

In the off-state or subthreshold region, the free-carrier concentration in the channel is low, resulting in a small dark current. Even a small number of photogenerated carriers entering the channel can cause a significant relative current change, which is beneficial for improving the photo-to-dark current ratio and weak light detection capability. In the on-state, the channel already has a high electron concentration, and the absolute value of the source-drain current is large. Illumination can further increase the carrier concentration or alter the local potential, but the relative change caused by light may be smaller because the dark current itself is high. The on-state is more suitable for situations requiring a larger readout current. Therefore, the gate voltage choice for MO TFT optoelectronic devices needs to be determined based on the specific application goals (Figure 2e).

The gate voltage also affects the separation, trapping, and release processes of photogenerated carriers. When the gate electric field changes the band bending at the channel and interface, the spatial distribution of photogenerated electrons and holes also changes accordingly. For n-type MO TFTs, a positive gate voltage typically promotes electron accumulation, making the channel more conductive; a negative gate voltage reduces the electron concentration, bringing the device closer to the off-state. If traps exist inside the channel or at the interface, the gate voltage can also change the probability of photocarriers being trapped or released, thereby affecting the current decay process after illumination. In PDs, this characteristic can be used to reduce dark current, enhance weak light response, and promote the release of residual charge through positive gate voltage pulses, allowing the device to return to its initial state faster, thus reducing the signal residue caused by PPC [11].

For OESs, the role of the gate voltage is not limited to adjusting the photoresponse intensity but can also participate in setting, modulating, and resetting the synaptic state. Light pulses can serve as write signals, causing channel conductance enhancement; gate voltage pulses can act as modulation, inhibition, or erasure signals, changing the conductance retention time and response to subsequent light stimulation. Through the combination of optical stimulation and electrical bias, MO TFTs can achieve optical input, electrical readout, and state modulation within the same device. Dual-gate or multi-terminal structures can further separate the processes of initial channel state setting, photoresponse modulation, and electrical resetting, but this also increases the complexity of the device structure and bias conditions. Therefore, the influence of gate voltage on photoresponse and charge relaxation processes is an important foundation for MO TFTs to possess both PD and OES functions.

### 2.3. Formation Mechanism of Persistent Photoconductivity

PPC is a common photoelectric phenomenon in MO TFTs, typically manifested as the device current remaining higher than the initial dark level after illumination ceases and decaying slowly over time. This phenomenon indicates that the change in carrier concentration or charge-trapping state induced by light can be maintained for a period after the light is turned off (Figure 2f). For MO semiconductors, PPC is generally associated with factors such as Neutral oxygen vacancy (V_O_), deep-level defects, interface states, surface adsorption, and local structural relaxation. Among these, the V_O_ is one of the most representative defects and is often considered a crucial source affecting the photoresponse, PPC, and OES memory behavior of MO TFTs.

In the mechanism related to V_O_, the key lies in the reversible transformation between their different charge states. The ground state oxygen vacancy is usually denoted as V_O_. Illumination or charge-trapping processes can convert it into ionized charged states (V_O_^+^, V_O_^2+^) [12,13,14]. This transformation changes the local charge distribution and channel electron concentration and may be accompanied by local lattice distortion. Since the charged oxygen vacancy state requires overcoming a certain energy barrier to return to the ground state, the conductive state after illumination is not immediately eliminated, thus forming PPC. The dynamic dissociation and recovery mechanism of oxygen vacancies is shown in Figure 2g.

When the incident photon energy exceeds the ionization energy ε of the V_O_ (ε being the energy difference between the ground state and the charged state), V_O_ is directly photo-excited and undergoes ionization. This process releases electrons, increasing the free electron concentration in the channel. The process can be represented as [12]

(1) $$V_{O}+hv\to V_{O}^{2+}+2e^{-}$$

When the photon energy is low, illumination may not directly excite oxygen vacancy ionization. However, V_O_ can still transform into charged states by capturing active holes near the valence band maximum. In this process, weakly bonded oxygen (O_wb_^2−^) and interstitial oxygen (O_i_) may participate in the defect transformation, forming metastable complex defects. This process can be represented as [15]

(2) $$V_{O}+O_{wb}^{2-}+2h^{+}\to V_{O}^{2+}/O_{i}+2e^{-}$$

This process shows that even if the incident photon energy is lower than that required for direct oxygen vacancy ionization, illumination may still promote oxygen vacancy ionization by inducing holes from other acceptor states. Thus, even under visible or near-visible light stimulation, wide-bandgap MO TFTs can exhibit a weak photoresponse and PPC through the indirect ionization process of V_O_.

The charged oxygen vacancy state is usually metastable. According to first-principles calculations, the recovery of the charged oxygen vacancy to the ground state requires overcoming a certain activation energy E_a_, and the defect-localized states (DLSs) need to recapture electrons. Because this recovery process is slow, the conductive state of the device after illumination does not recover immediately, manifesting as a slow decay of the source-drain current. The recovery process of oxygen vacancies can be represented as

(3) $$V_{O}^{2+}\;or\;V_{O}^{2+}/O_{i}+2e^{-}+E_{a}\to V_{O}\;or\;V_{O}+O_{wb}^{2-}$$

Thus, the strength of PPC is closely related to the stability of the charged oxygen vacancy state and the energy barrier of the recovery process. When the activation energy E_a_ is large, the time required for the charged oxygen vacancy to return to the ground state is longer, the PPC effect is more pronounced, and the current relaxation time after illumination is longer. For PDs, this slow recovery process causes response lag and signal residue; for OES devices, this process can be used to simulate state retention and forgetting behavior after optical stimulation. Therefore, V_O_ not only affect the responsivity of MO TFTs but also determine their temporal response characteristics in PD and OES applications.

Besides V_O_, interface states, surface adsorption, and other deep-level defects can also influence PPC. Interface traps between the channel and the gate dielectric or capping layer can trap photogenerated charges and continuously modulate the channel conductance through a gate-like effect. Oxygen and moisture adsorption/desorption processes on the surface of metal oxides exposed to air can further slow down the charge recovery after illumination. However, compared to these auxiliary factors, the dynamic dissociation and recovery of V_O_ better represent the intrinsic defect source of PPC in MO TFTs. Therefore, when analyzing the slow recovery of PDs and the memory retention of OES devices, it is necessary to focus on factors such as V_O_ concentration, defect energy levels, charged state stability, and recovery activation energy.

### 2.4. Functional Duality of Persistent Photoconductivity

PPC has different functional significance in different optoelectronic devices. For PDs, the ideal state is typically that the device responds quickly when light arrives and recovers rapidly when light ceases. Excessive PPC prolongs the fall time, causing signal residue, making it difficult for the device to accurately distinguish consecutive light pulses or rapidly changing optical signals. In image sensing and dynamic light detection, PPC can also cause signals from the previous frame or previous illumination to persist into subsequent readouts, affecting temporal resolution and image refresh rate.

Therefore, whether PPC is advantageous depends not only on its strength but also on whether the current decay time matches the readout time window of the target application. For high-speed photodetection, the current should recover as quickly as possible after illumination, and the PPC decay time typically needs to be much shorter than the light pulse interval or the signal modulation period. For frame-based image sensing, the residual photocurrent should decay sufficiently before the next frame is read; therefore, the PPC timescale must be shorter than the frame interval, or, at least, frame-to-frame crosstalk must be reduced through gate voltage reset and circuit calibration.

Therefore, in PDs, PPC usually needs to be suppressed or its recovery accelerated. Common methods include reducing deep-level defect density [16], improving film densification [17], optimizing V_O_ concentration [18,19], introducing interface passivation layers [20], reducing surface adsorption effects [17], and using pulsed gate voltage reset [11]. The goal is not to completely eliminate all defects but to reduce uncontrollable long-term charge retention, allowing the photocurrent to return to dark levels within a reasonable time. For high-sensitivity detectors, a balance between responsivity and response speed is also needed, as high responsivity often comes from trap-assisted gain or long carrier lifetime, which can also enhance PPC.

For OES devices, PPC can be exploited as a functional source. The slow current decay after illumination can simulate memory retention and forgetting behavior in biological synapses. If the current drops quickly after light stimulation, the device exhibits short-term memory characteristics; if the current remains at a high level for a longer time, it can exhibit long-term memory characteristics. When consecutive light pulses are applied, the residual conductance from the previous pulse has not completely disappeared, and the subsequent pulse further increases the channel conductance, leading to a gradual enhancement of the postsynaptic current. This process can simulate paired-pulse facilitation, the transition from short-term plasticity to long-term plasticity, and synaptic weight updates.

Unlike PDs, OES devices require a conductance relaxation process that persists for a certain time scale. When used for time filtering or motion tracking, the PPC time constant can be designed to range from milliseconds to seconds, enabling the device to generate history-dependent responses to successive light stimuli. For short-term plasticity and short-term memory, the post-illumination conductance retention typically needs to span the time interval between consecutive stimuli, corresponding to decay processes ranging from seconds to minutes. If the goal is long-term memory or relatively stable synaptic weight storage, a longer conductance retention period is required, which can extend to minutes, hours, or even longer. Thus, OES devices do not simply aim for the strongest and longest-lasting PPC; rather, they require selecting an appropriate decay time window based on functions such as time filtering, short-term memory, or long-term weight storage.

**Figure 3:**
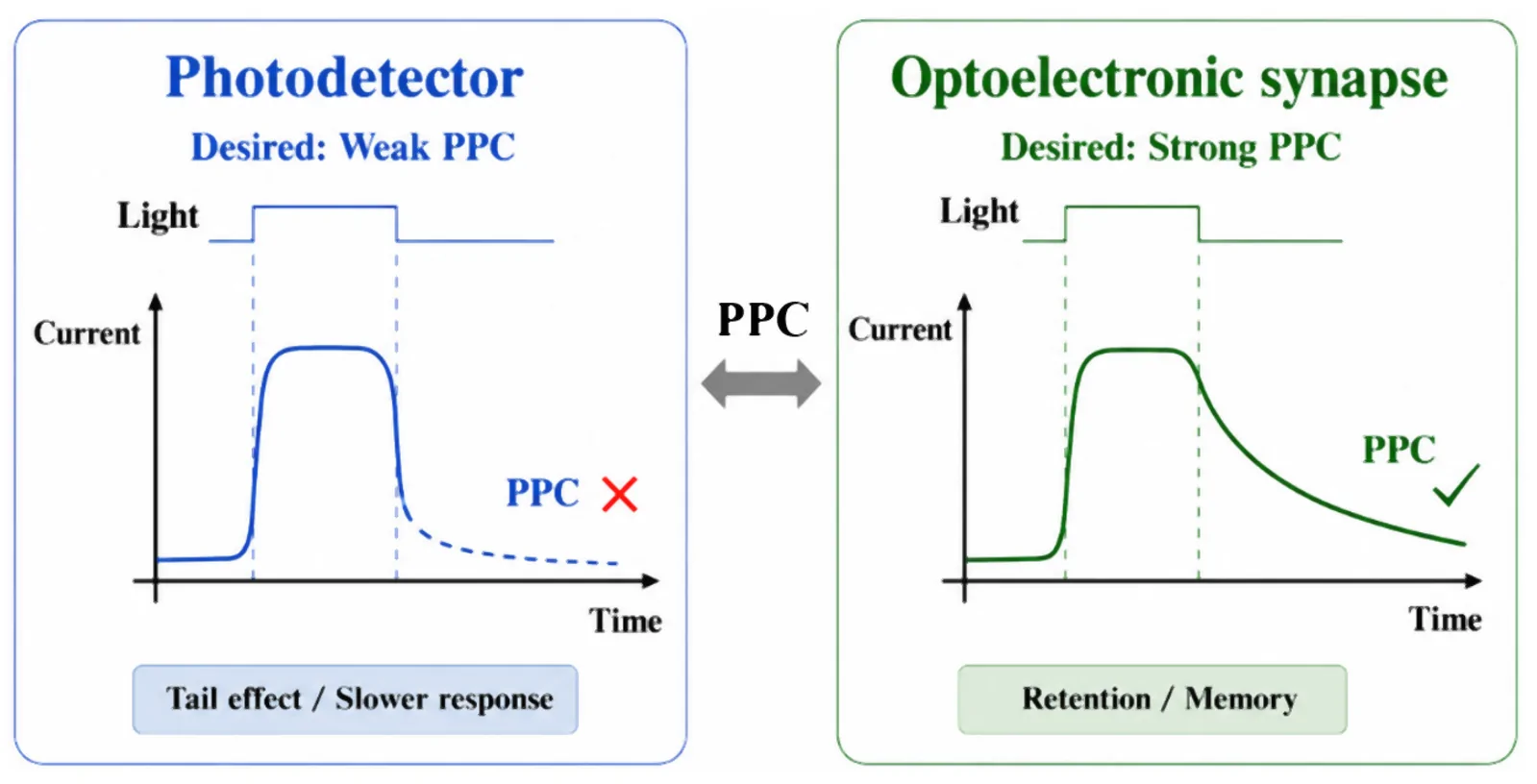
Performance divergence: weak PPC with fast response for photodetectors vs. strong PPC with long retention for optoelectronic synaptic devices.

Thus, PPC should not be simply defined as advantageous or disadvantageous. For PDs, it mainly manifests as slow recovery and signal residue, needing suppression through material, interface, and bias control. For OES devices, it can provide state retention and time-dependent conductance modulation, requiring controllable design through trap depth, defect density, interface charges, and gate control conditions. The difference between the two types of devices does not lie in completely different physical processes but in the different time scales and controllability requirements for the same carrier relaxation process (Figure 3).

This functional transformation also explains why MO TFTs can be used for both PD and OES research. The instantaneous current change caused by light can serve as a detection signal, while the residual conductance and slow decay after illumination can serve as memory signals. Under different bias conditions, optical stimulation methods, and readout time windows, the same device may exhibit characteristics that favor either fast detection or state retention. Therefore, PPC is an important physical process connecting PDs and OES and is the foundation for subsequent realization of multi-function synergy in single devices and in-sensor computing at the array level.

### 2.5. Summary of This Chapter

This chapter discussed the fundamental photoelectric physical processes in MO TFTs. The photoelectric behavior of the device is jointly determined by material absorption, channel transport, defect, and interface processes, and is further influenced by applied bias conditions.

The gate voltage modulation in TFTs provides an additional means of controlling the photoresponse. By changing the initial channel state and interface electric field, the gate voltage can affect the dark current, the photocurrent, and the charge release process. For PDs, the gate voltage setting needs to be selected based on the target application. For OES devices, the gate voltage can also be used to adjust conductance retention and state reset, enabling optical writing, electrical reading, and electrical modulation within the same TFT platform.

PPC has different functional implications in the two types of devices. PPC in metal oxides is closely related to the dynamic ionization and recovery of V_O_, and its strength and time scale are also influenced by interface traps, surface adsorption, and deep-level defects. For PDs, PPC typically manifests as response lag and signal residue; for OES devices, moderate PPC can be transformed into memory retention and weight modulation. Therefore, the control of PPC should not only aim for suppression or enhancement but should be reasonably regulated according to device functional requirements.

## 3. MO TFT Photodetector Device Research

The previous chapter discussed the fundamental photoelectric physical processes in MO TFTs. For PDs, these physical processes need to be further translated into evaluable device performance metrics, including the current change induced by light, the response/recovery process, and long-term operational stability.

Therefore, this chapter starts from the performance evaluation of PDs and discusses the research progress of MO TFT PDs. The content first revolves around the basic operating modes and key performance indicators, then reviews related research progress according to the response band to illustrate the performance characteristics and development issues of detectors in different bands.

### 3.1. MO TFT PD Overview and Key Performance Indicators

A PD is a device that converts incident optical signals into readable electrical signals, used to obtain information such as light intensity, wavelength, irradiation time, and spatial distribution. Depending on the device structure and signal conversion method, photodetectors typically include photoconductive [21], photovoltaic [22], and phototransistor [23] types. Among them, the MO TFT PD belongs to the phototransistor type, where the photoresponse is read through changes in source-drain current and is influenced by illumination conditions, gate voltage, source-drain voltage, and device structure.

In MO PDs, the research focus is not only on whether the material can absorb light of a specific wavelength but also on whether the optical signal can be converted into a stable, distinguishable, and repeatedly readable current output under the TFT operating state. As the application band gradually expands from UV detection to visible light, NIR, and broadband spectra, device design has also extended from single oxide channels to composite channels, heterojunctions, photosensitive layer modification, and multi-terminal control structures. Correspondingly, performance evaluation needs to shift from a single photoresponse intensity to a comprehensive consideration of dark current background, response speed, spectral selectivity, and operational stability.

#### 3.1.1. Responsivity

Light responsivity (*R*) is the magnitude of the output electrical signal (current or voltage) generated by a photodetector per unit incident light power, with units of A/W or V/W. It is typically expressed as

(4) $$R=\frac{I_{photo}-I_{dark}}{PS\;}$$

where *P* is the incident optical power density per unit area on the detector (W/m^2^), and *S* is the effective incident area (m^2^). Responsivity reflects the efficiency of the PD in converting optical signals into electrical signals and is one of the key parameters for evaluating detector performance. Its value depends on factors such as the detector’s material, structure, operating wavelength, and external bias conditions [24,25].

#### 3.1.2. Photo-to-Dark Current Ratio

Dark current (*I_dark_*) is the current output of a PD under a certain bias voltage in dark conditions. In practical applications, the PD will have an output current even when no light signal is received, which is the dark current with the unit A. This value is considered a major source of noise; the smaller the value, the better the device performance. Photocurrent (*I_photo_*) refers to the output current of the photodetector under a certain bias voltage when illuminated with light of a specific wavelength. The magnitude of the photocurrent depends on the incident optical power. The photo-to-dark current Ratio (PDCR) is the ratio of photocurrent to dark current. Generally, the higher the PDCR of a photodetector, the better [26,27,28].

(5) $$PDCR=\frac{I_{photo}}{I_{dark}}$$

However, this metric is susceptible to variations in the absolute values of the dark current and the incident light power density. When there are significant differences in the light intensity used across different studies, simply comparing the PDCR may not adequately reflect responsivity per unit of light power. Therefore, the normalized photocurrent-to-dark-current ratio (NPDR) is also commonly used to supplement the evaluation of responsivity. Its expression can be written as

(6) $$NPDR=\frac{PDCR}{P}$$

Here, *P* is the incident optical power density. NPDR can be used to compare the relative light response capabilities of devices under different light power conditions. A higher NPDR typically indicates that a device is capable of producing a significant current change in response to weak light against a low dark-state background and therefore has reference value for weak-light detection and image sensing applications.

#### 3.1.3. External Quantum Efficiency

External Quantum Efficiency (EQE) characterizes the efficiency of converting incident photons into collectable charges in the external circuit, i.e., the number of effective charge carriers corresponding to each incident photon. A higher EQE indicates a stronger photoelectric conversion or gain capability of the device. EQE can usually be converted from responsivity:

(7) EQE=hcR/qλ×100%

#### 3.1.4. Specific Detectivity

Specific detectivity (D*) is used to characterize the ability of a PD to detect weak optical signals. It is a performance indicator that comprehensively considers the device’s responsivity, noise level, and effective photosensitive area, emphasizing the device’s ability to identify weak light signals against a noise background. Under the approximation where dark current shot noise dominates, the specific detectivity is typically expressed as

(8) $$D^{*}=\frac{R\sqrt{S}}{\sqrt{2qI_{dark}}}$$

The common unit for D* is Jones. Higher responsivity and lower dark current both contribute to improving the specific detectivity. Increased responsivity means the device generates a stronger electrical signal for the same incident optical power, while lower dark current helps reduce the noise background, thereby enhancing weak-light resolution capability.

It should be noted that the above formula is based on the assumption that dark current shot noise is dominant. When 1/f noise, generation-recombination noise, random telegraph noise, or external readout noise dominates in a device, using this approximate formula alone may overestimate the device’s actual low-light detection capability. Therefore, when evaluating MO TFT PDs, the calculation of D* should be based on measured noise current whenever possible, or the noise model used should be clearly specified. In this context, noise equivalent power (NEP) can be used to describe the minimum resolvable incident light power when the signal-to-noise ratio is 1; it is typically related to the responsivity and noise current. In practical evaluations, D* should be considered in conjunction with NEP, noise spectral density, and the test bandwidth. Particularly under conditions of low-frequency readout, long integration times, and array imaging, the detectivity derived from the actual noise spectrum more accurately reflects the device’s true performance in practical applications.

#### 3.1.5. Response Time

The response time of a photodetector refers to the speed at which the detector reacts to changes in the incident light signal. It typically includes the rise time (*t_rise_*) and the fall time (*t_fall_*). Response time is an important parameter for measuring dynamic performance, reflecting the time required for the detector to reach a stable output value after receiving an optical signal. Generally, it refers to the time taken for the output electrical signal to rise from 10% to 90% of its stable value after receiving a light pulse (rise time), and the time taken to fall from 90% to 10% of the stable value after the light pulse ceases (fall time). Typically expressed as

(9) $$t_{rise}=t_{90\%}-t_{10\%}$$

(10) $$t_{fall}=t_{10\%}-t_{90\%}$$

The shorter the response time, the stronger the photodetector’s ability to react to rapidly changing optical signals, suitable for high-frequency or high-speed optical communication systems. Excessively long response time can cause signal distortion and limit the application scenarios of the detector. It is primarily related to the carrier lifetime, carrier recombination time within the material, and the device structure [29,30].

#### 3.1.6. Rejection Ratio

The rejection ratio (RR) is the ratio of the detector’s responsivity at the target wavelength (usually the operating wavelength) to its responsivity at a non-target wavelength (usually an interfering wavelength). It is used to measure the detector’s ability to distinguish optical signals of different wavelengths, particularly the ability to suppress interference from non-target wavelengths in multi-wavelength or broadband light-source environments. The rejection ratio is an important application parameter, determining suitability for different wavelength range requirements, suppressing multi-wavelength interference, extracting target signals in complex light environments, and improving detection accuracy [31,32]. In this review, the rejection ratio is expressed as R254/R280, representing the ratio of responsivity under 254 nm illumination to that under 280 nm illumination.

#### 3.1.7. Noise

Noise is an important factor limiting the weak-light resolution capability of photodetectors, typically manifested as current or voltage fluctuations without effective optical signal input. For MO TFT PDs, noise may originate from dark current shot noise, channel transport fluctuations, interface traps, and the measurement circuit. Under the approximation where dark current shot noise dominates, the noise current can be expressed as

(11) $$i_{n}=\sqrt{2qI_{dark}}$$

Therefore, lower dark current helps reduce the noise background and improve specific detectivity and weak-light detection capability. For TFT PDs used in image sensing and low-light detection, it is also necessary to further consider the effects of low-frequency noise on the minimum detectable signal, image uniformity, and long-term readout stability.

In addition to shot noise associated with dark current, low-frequency noise is also a key factor affecting the actual performance of MO TFT PDs. Under low-frequency or quasi-static readout conditions, 1/f noise, generation-recombination noise, and random telegraph noise may become the primary noise sources, further affecting the minimum detectable signal, detection efficiency, and image uniformity. For MO TFTs, low-frequency noise is typically associated with channel defects, oxygen vacancies, traps at the gate dielectric/channel interface, and carrier capture–release processes caused by environmental adsorption. Previous studies have shown that low-frequency noise in a-IGZO TFTs primarily manifests as 1/f noise, the intensity of which is closely related to the quality of the active layer, the distribution of trap states, and the gate dielectric interface [33,34,35]. Therefore, the noise evaluation of MO TFT PDs should not rely solely on estimates of dark current shot noise but should also incorporate analysis of the low-frequency noise spectrum. Through defect control, interface passivation, gate dielectric optimization, and circuit-level noise suppression methods, trap-related current fluctuations can be reduced, thereby improving the reliability of devices in low-light detection, image sensing, and optoelectronic synapse arrays.

#### 3.1.8. Power Consumption and Stability

Operating power consumption is also a key indicator for evaluating the practical application potential of MO TFT PDs. Operating power consumption is also a key indicator for evaluating the practical application potential of MO TFT PDs. Power consumption is primarily related to the source-drain voltage, source-drain current, and gate leakage current, with the source-drain channel current typically accounting for the largest portion. A higher bias voltage helps enhance carrier collection and increase the photocurrent, but it may also increase dark current, power consumption, noise, and bias stress. For large-area arrays, flexible vision sensing, and edge computing applications, the power consumption of individual pixels accumulates to form the system’s total power consumption. Therefore, low-power operation must be considered in conjunction with long-term stability, with particular attention given to the ability to maintain photoresponse under low-bias conditions, the reliability of repeated readouts, and performance drift caused by bias stress.

Stability describes the ability to maintain reliable output under repeated illumination, applied bias, and environmental changes. Stability typically includes illumination-cycling stability, bias stability, photo-bias stability, and environmental stability. Illumination-cycling stability focuses on whether baseline drift, photocurrent decay, or incomplete recovery occurs during multiple light on/off cycles. Bias stability mainly reflects changes in dark current, threshold voltage (Vth), and response amplitude under gate voltage or source-drain voltage stress. Photo-bias stability further examines device drift under the combined effect of illumination and electric field. Environmental stability relates to external factors such as oxygen, moisture, and temperature. Since these factors directly affect the dark current background, photoresponse repeatability, and long-term readout reliability, stability is an important aspect of evaluating the practical application potential of MO TFT PDs.

### 3.2. MO TFT PDs Categorized by Response Band

#### 3.2.1. DUV and SBUV Detection

DUV and SBUV detection is an early direction that garnered attention in MO TFT PDs. SBUV typically refers to the 200–280 nm band. Since this band is strongly absorbed by the ozone layer in the Earth’s atmosphere, the background light on the ground is weak, making it valuable for applications such as flame warning, space communication, high-voltage corona discharge detection, environmental monitoring, and UV imaging. For such detectors, materials need to have efficient absorption in the DUV region while maintaining low responsivity to visible and near-UV background light. Wide-bandgap and ultra-wide-bandgap metal oxide semiconductors typically have absorption edges in the UV or DUV region, offering good spectral selectivity, chemical stability, thermal stability, thin-film fabrication, and large-area integration advantages, making them important material systems for DUV and SBUV TFT PDs.

In DUV and SBUV photodetection research, Ga_2_O_3_ is often used to construct photodetectors because its absorption edge matches the SBUV band relatively well. Ga_2_O_3_ has an ultra-wide bandgap of approximately 4.8–4.9 eV, and its intrinsic absorption edge aligns reasonably with the SBUV band, allowing the suppression of visible and near-infrared background interference without relying on external filters.

**Figure 4:**
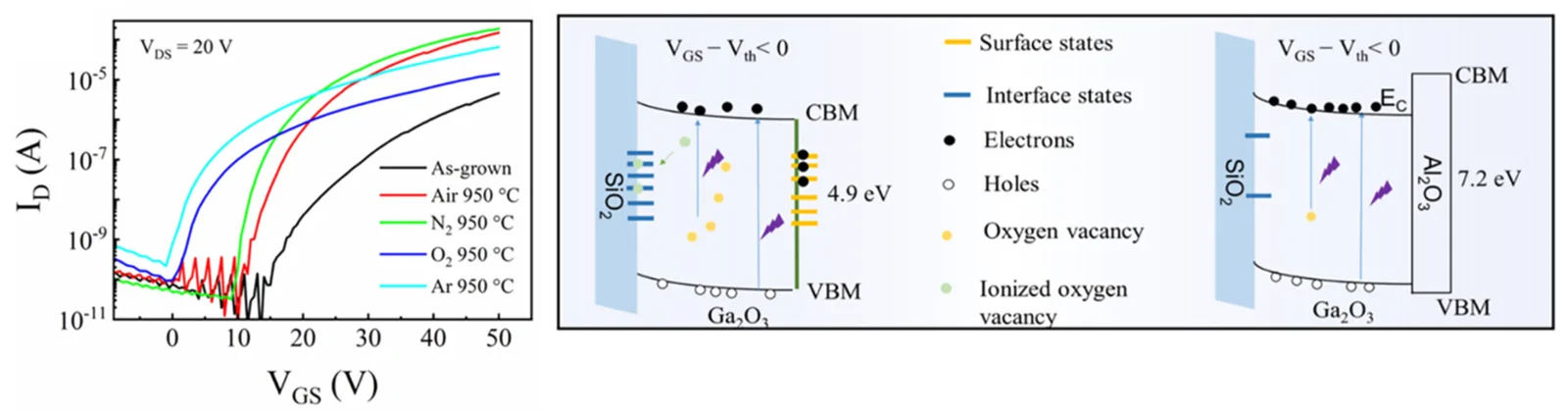
Transfer characteristic curves of Ga_2_O_3_ TFTs before and after annealing under various atmospheres and energy band structure diagrams for Ga_2_O_3_ TFTs incorporating an Al_2_O_3_ passivation layer [36]. Reproduced with permission from Ref. [36]. Copyright © 2026 American Chemical Society.

In Ga_2_O_3_ TFT PDs, the crystalline characteristics, defect states, and interface quality of the film collectively affect the dark current, photoresponse, and recovery process. Among crystalline Ga_2_O_3_, β-Ga_2_O_3_ is commonly used in DUV and SBUV detector research due to its good thermal stability, ultra-wide bandgap, and relatively mature material research foundation. Xu Haiyang’s group effectively passivated defects in β-Ga_2_O_3_ thin films through high-temperature nitrogen annealing and atomic-layer-deposition alumina encapsulation. The treated transistors showed an electron mobility increase of more than 27 times and a SBUV responsivity increase of more than 94 times [36] (Figure 4).

**Figure 5:**
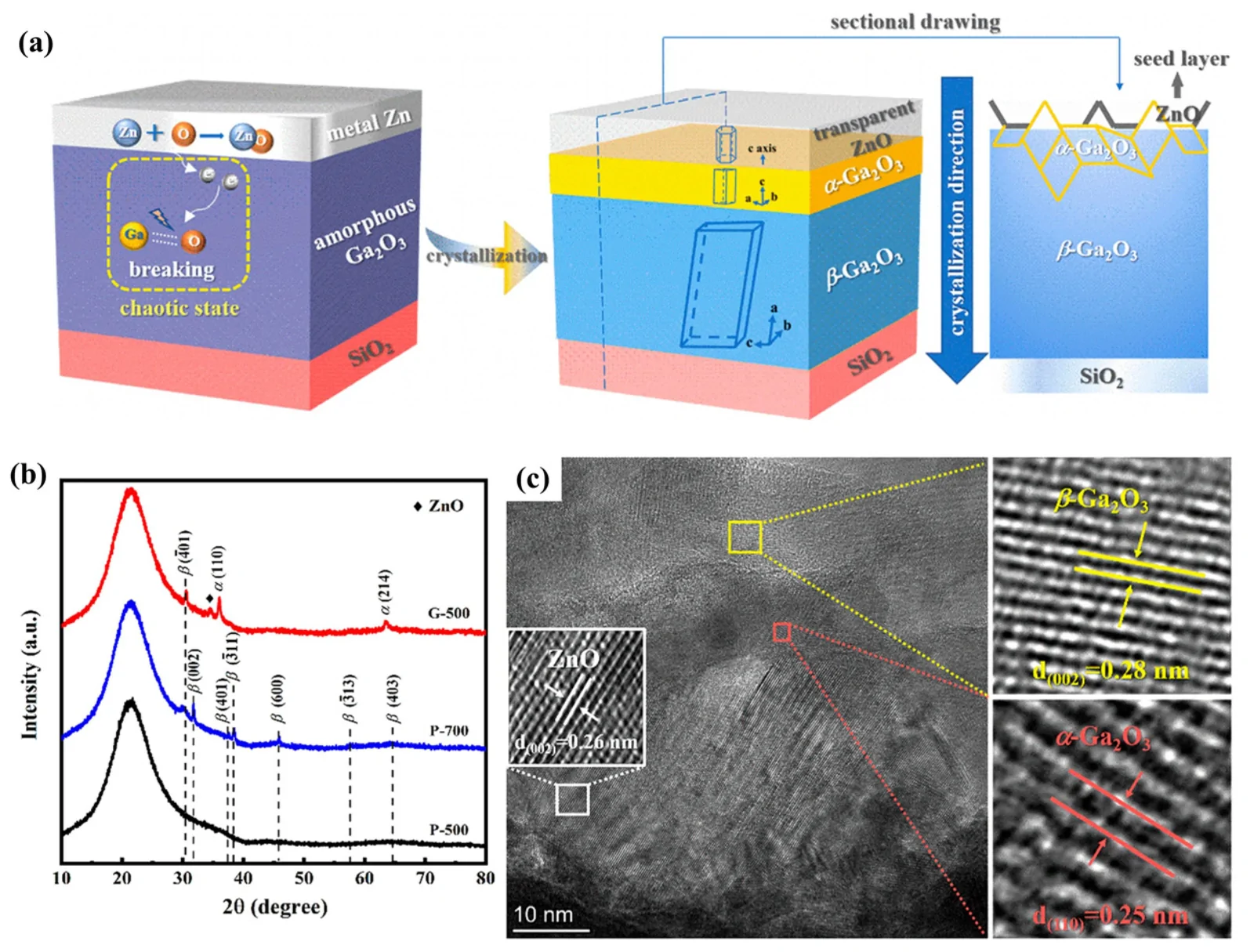
(**a**) Schematic diagram of Zn metal-induced low-temperature crystallization of Ga_2_O_3_. (**b**) XRD results and (**c**) TEM results of the low-temperature crystallization [38]. Reproduced with permission from Ref. [38]. Copyright © 2022 American Chemical Society.

**Figure 6:**
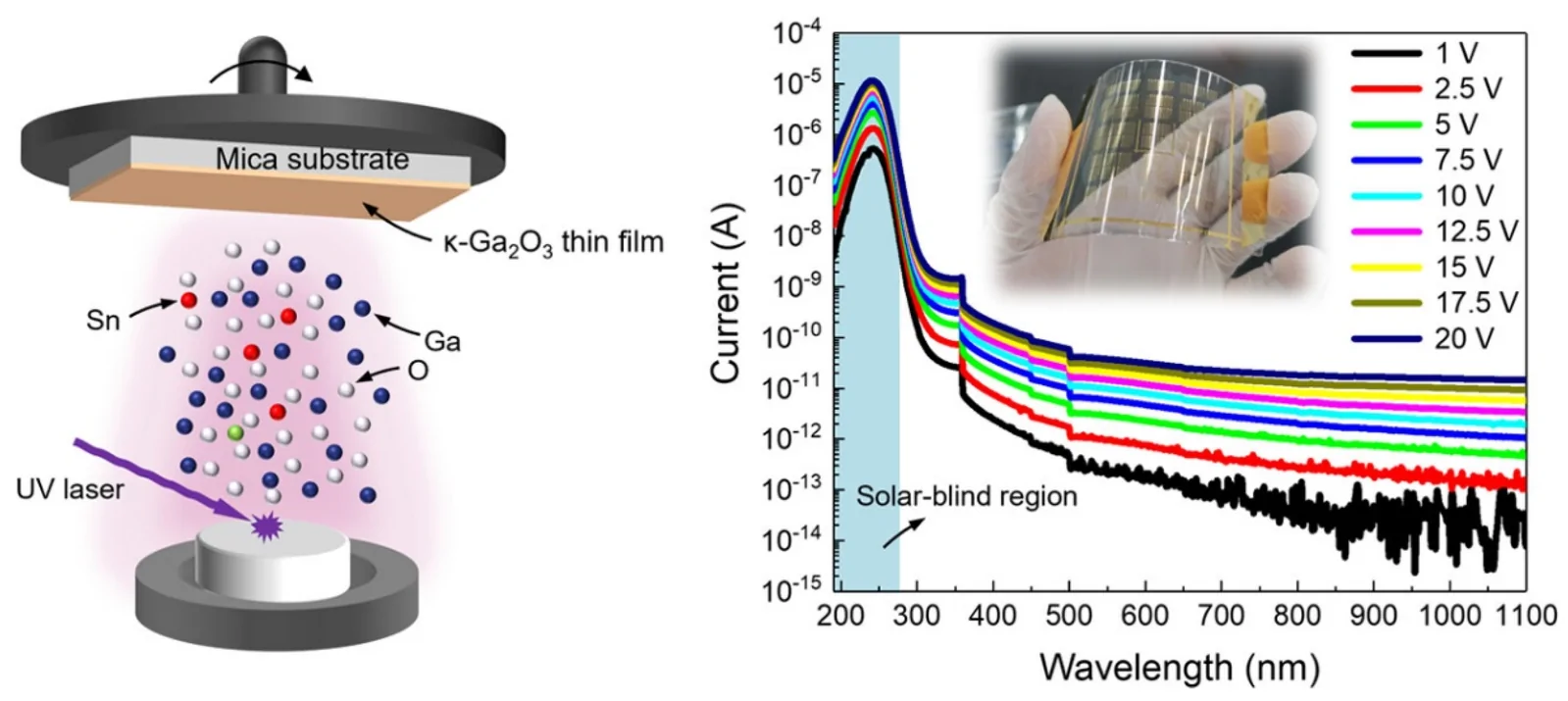
Schematic diagram of κ-Ga_2_O_3_ PD preparation and photocurrent in the 190–1100 nm wavelength range [39]. Reproduced from Ref. [39] under the terms of the Creative Commons Attribution-NonCommercial-NoDerivatives 4.0 International License (CC BY-NC-ND 4.0).

**Figure 7:**
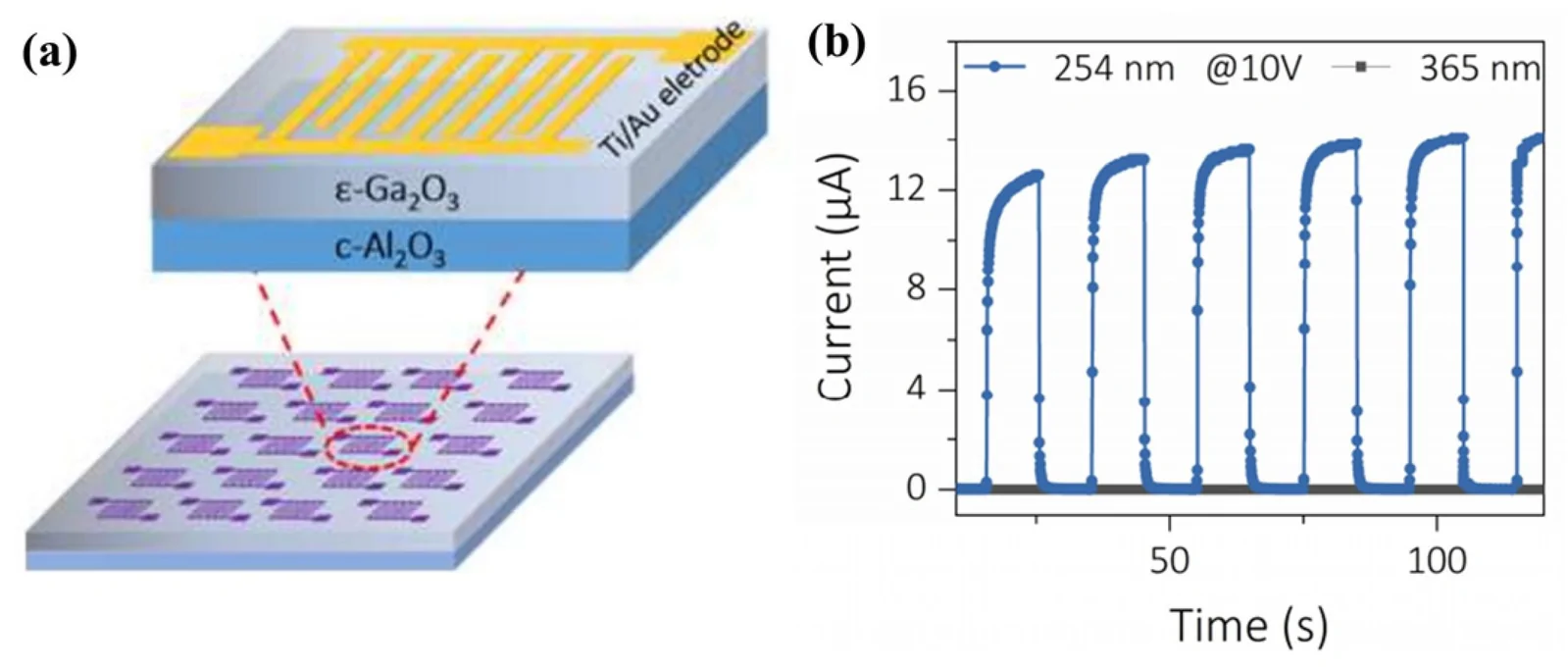
(**a**) Schematic diagram of ε-Ga_2_O_3_ PD preparation and (**b**) I-T curves under 254 nm and 365 nm illumination [40]. Reproduced from Ref. [40] under the Creative Commons Attribution 4.0 International License (CC BY 4.0).

Higher crystalline quality benefits channel transport and reduces defect-related responses, but its preparation typically requires high temperatures, and substrate matching and gate dielectric interface quality affect the process compatibility of TFTs [37]. Pei et al. achieved the low-temperature crystallization of gallium oxide by Zn induction, successfully lowering the crystallization temperature of Ga_2_O_3_ by 200 °C, realizing high-quality low-temperature crystallization (Figure 5). The obtained detector achieved a SBUV/visible rejection ratio as high as 2 × 10^5^ [38]. Besides the β phase, metastable phases like κ-Ga_2_O_3_ [39] (Figure 6) and ε-Ga_2_O_3_ [40] (Figure 7) have also been studied for DUV detection. Their wide bandgap and different crystal structures offer more possibilities for bandgap engineering, epitaxial growth, and heterojunction interface design, but phase stability, low-temperature preparation, and large-area uniformity still require further work.

**Figure 8:**
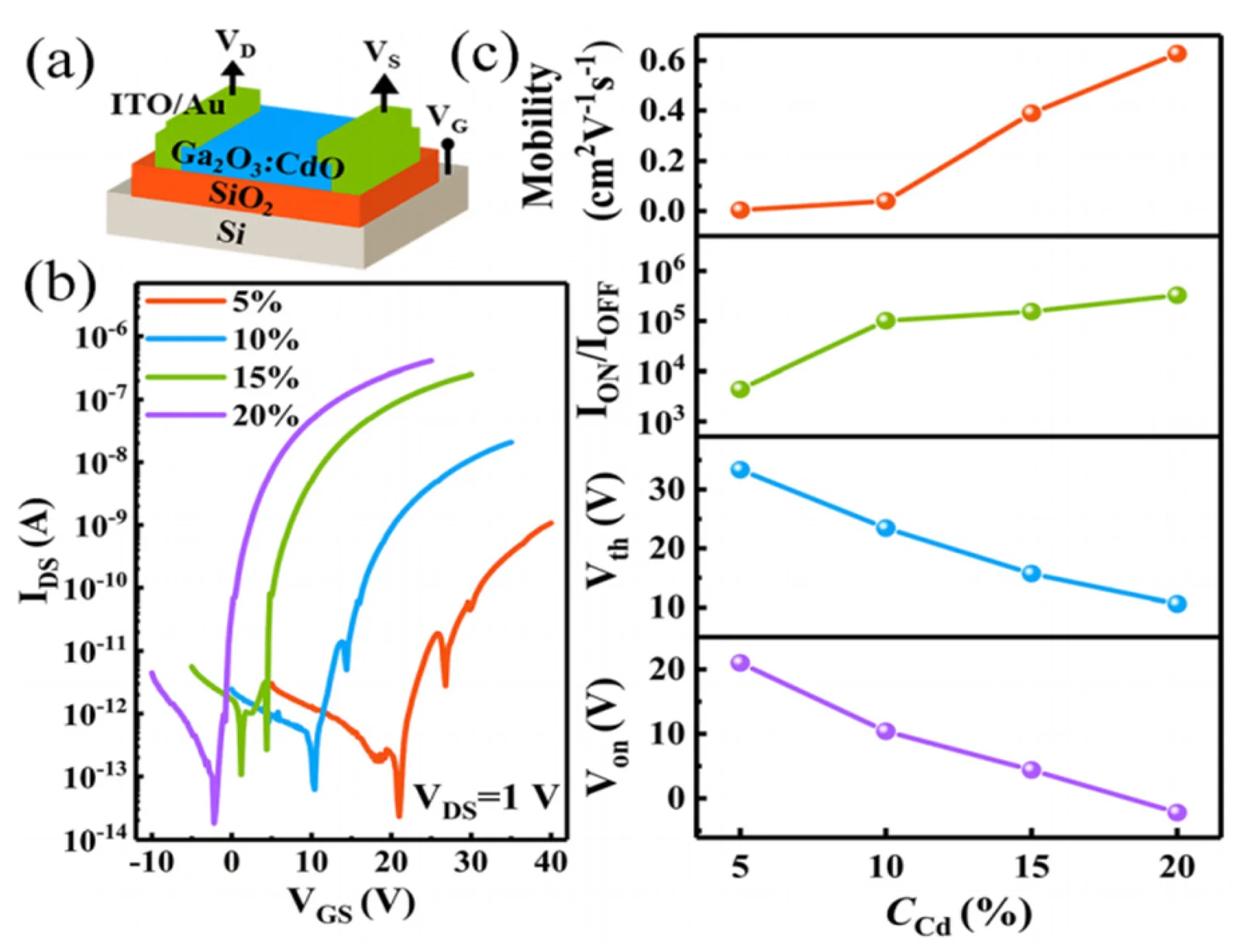
(**a**) Schematic structure, (**b**) transfer curves and (**c**) electrical parameters of Ga_2_O_3_:CdO TFT photodetector [41]. Reproduced with permission from Ref. [41]. Copyright © 2020 AIP Publishing.

**Figure 9:**
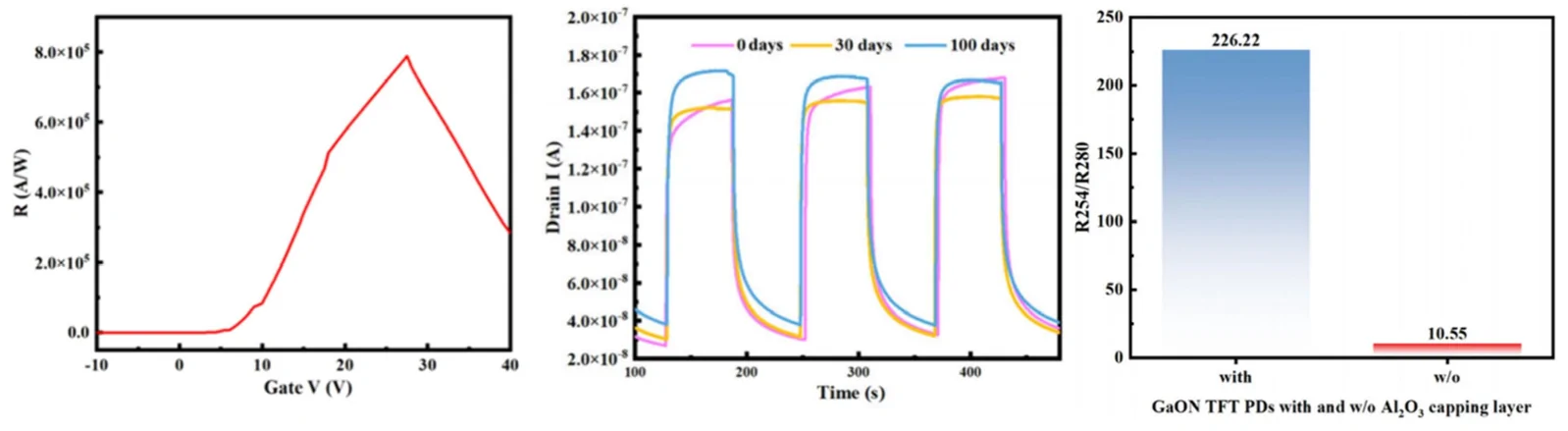
Responsivity, environmental stability, and rejection ratio of Ga_2_O_3_/Al_2_O_3_ PDs [45]. Reproduced with permission from Ref. [45]. Copyright © 2026 American Chemical Society.

**Figure 10:**
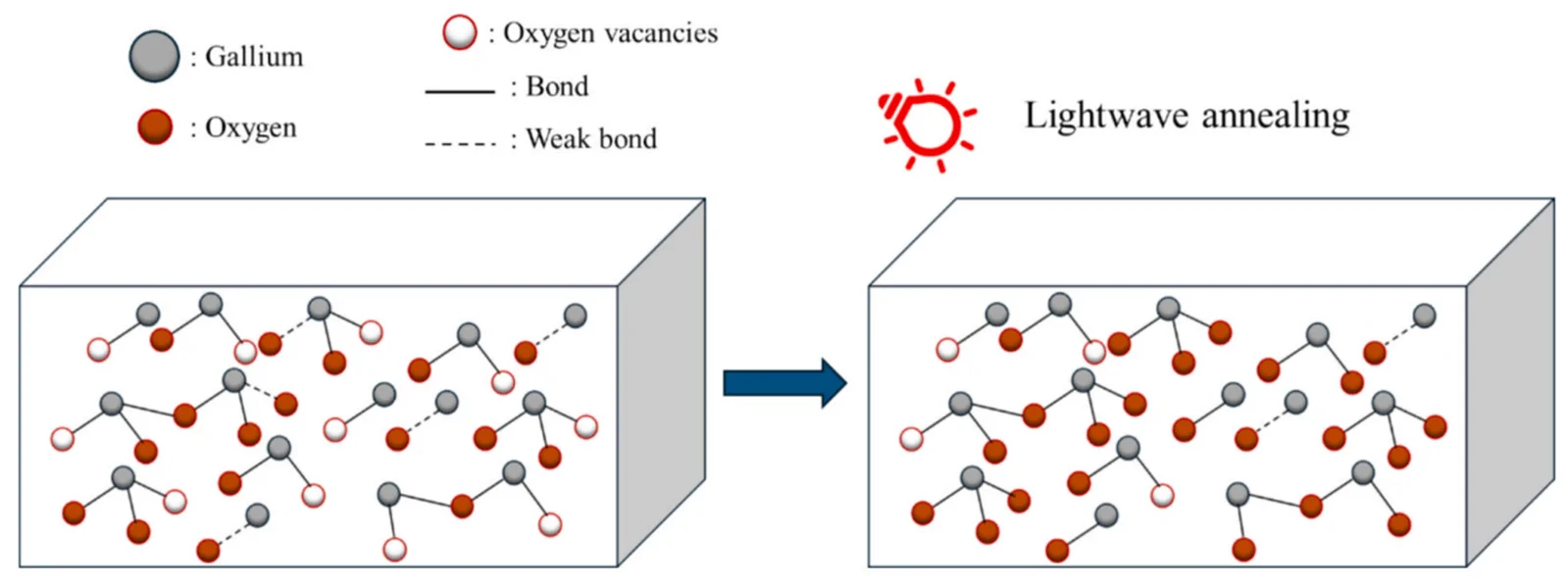
Schematic representation of the microstructural evolution of a-Ga_2_O_3_ thin films during lightwave annealing process [46]. Reproduced with permission from Ref. [46]. Copyright © 2026 Elsevier.

In contrast, amorphous Ga_2_O_3_ is more suitable for low-temperature and large-area preparation. Its structure lacks long-range order and is less dependent on lattice mismatch and substrate orientation, thus possessing high defect tolerance and being capable of forming relatively uniform films on large-area or flexible substrates like glass and polymers. In amorphous Ga_2_O_3_, cation doping can effectively modulate its photoelectric properties. Xiao et al. effectively controlled the optical bandgap of amorphous Ga_2_O_3_:CdO thin films and the electrical characteristics of TFTs by varying the Cd content. The fabricated amorphous Ga_2_O_3_:CdO TFT DUV detector achieved a high responsivity of 2.17 A/W and a UV/visible rejection ratio of 1.88 × 10^4^ [41] (Figure 8). In addition to composition control, physical parameters in the film deposition process can also significantly affect defect states. Pei et al. adjusted the substrate bias during magnetron sputtering, effectively controlling the composition ratio and defects of a-GaO_x_ films, increasing the device’s responsivity by 460 times compared to the unoptimized process [42]. Lei et al. achieved in situ H incorporation in GaO_x_, finding that hydrogen-related unstable defects introduced by water vapor were significantly reduced after vacuum annealing, thereby improving the film’s density and microstructural order, ultimately increasing device responsivity from 44.05 A/W (pre-hydrogen) to 832.6 A/W, an order-of-magnitude leap [43]. To deeply reveal the nature of these defect states, Cheng et al. used TCAD to quantitatively fit the transient photocurrent curves of gallium oxide photodetectors, extracting band tail state and deep-level defect state parameters, establishing a comprehensive analysis method combining experimental fabrication and physical TCAD modeling [44]. Based on the above defect control and physical understanding, Ren et al. introduced Al for multifaceted comprehensive optimization of amorphous GaO_x_, covering low-temperature crystallization, optical modulation, and stability enhancement. The optimized device achieved an ultrahigh responsivity of 7.8 × 10^5^ A/W and maintained performance stability even after 100 days of air exposure (Figure 9) [45]. Tang et al. used lightwave annealing technology for rapid and efficient post-treatment of amorphous GaO_x_ films, effectively reducing defect state density. Phototransistors based on this achieved a specific detectivity exceeding 10^18^ Jones, particularly suitable for weak ultraviolet signal detection (Figure 10) [46]. Local disorder and defect states in the amorphous structure limit carrier transport and cause PPC and recovery delays. Therefore, performance optimization of amorphous Ga_2_O_3_ TFT detectors still needs to focus on V_O_, deep-level defects, and interface quality, balancing low-temperature processing advantages with stable photoelectric response.

**Figure 11:**
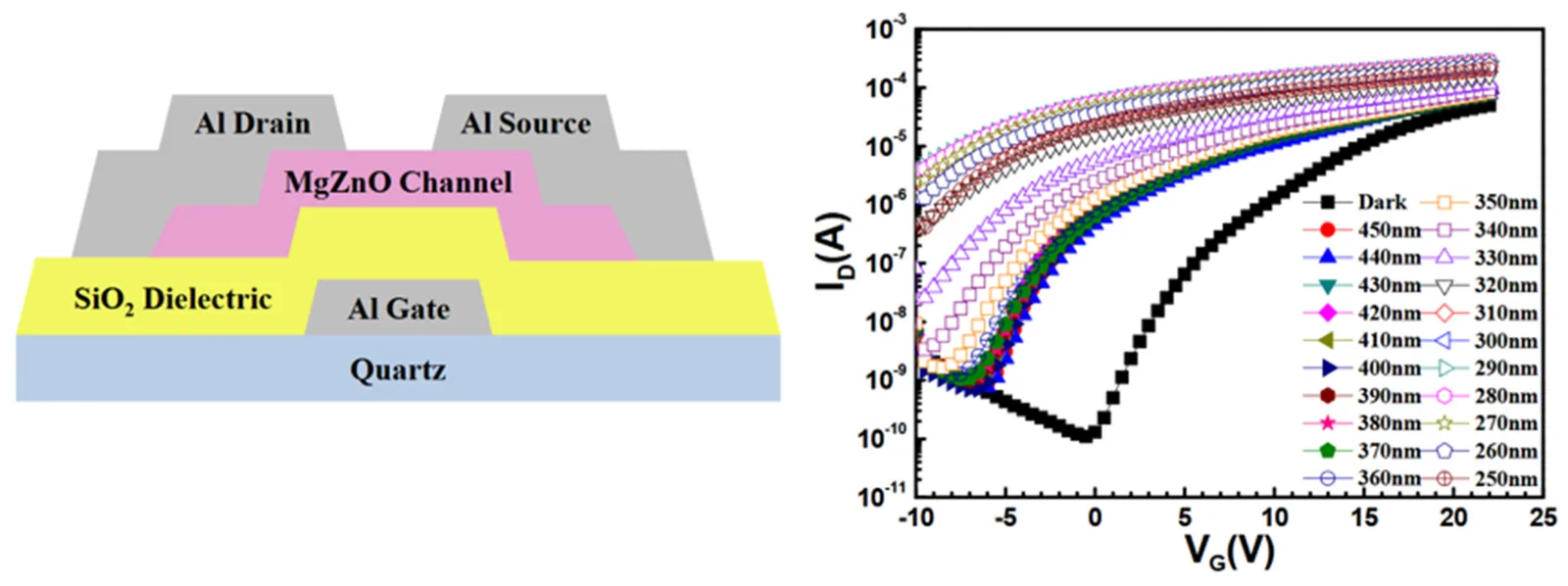
Schematic of the MgZnO PD structure and transfer curves under different wavelengths of illumination [49]. Reproduced from Ref. [49] under the Creative Commons Attribution 4.0 International License (CC BY 4.0).

In addition to Ga_2_O_3_, ZnO and its alloys and composite oxides are also common material systems for UV photodetectors. ZnO has a bandgap of about 3.3 eV, with intrinsic absorption mainly in the near-UV region, making it more suitable for general UV or visible-blind UV detection, but difficult to directly meet strict solar-blind detection requirements [47]. To shift the response range to shorter wavelengths, Mg alloying to form MgZnO is widely used for bandgap tuning. As Mg content increases, the absorption edge can blue-shift towards the DUV direction, enhancing solar-blind selectivity [48,49] (Figure 11). However, high Mg content may cause phase separation, reduced crystal quality, and limited carrier transport, so a balance is needed between bandgap widening and thin-film electrical properties [50]. Similar to MgZnO, Ga-containing composite oxides like ZnGaO and ZnGa_2_O_4_ can also adjust the absorption edge, defect distribution, and carrier transport characteristics through composition variation [51]. Fan et al. reported a ZnGaO TFT SBUV PD, optimizing device performance by adjusting the ZnO cycle ratio. At 40% ZnO cycle ratio, an ultra-low dark current of approximately 4.5 × 10^−13^ A, an optical on/off ratio of about 2.2 × 10^7^, and a responsivity of about 34.31 A/W were obtained [52]. ZnO-based alloys and composite oxides offer a material tuning path for DUV and SBUV detection different from single Ga_2_O_3_, but phase stability, mobility, and defect-related stability still need further optimization.

**Figure 12:**
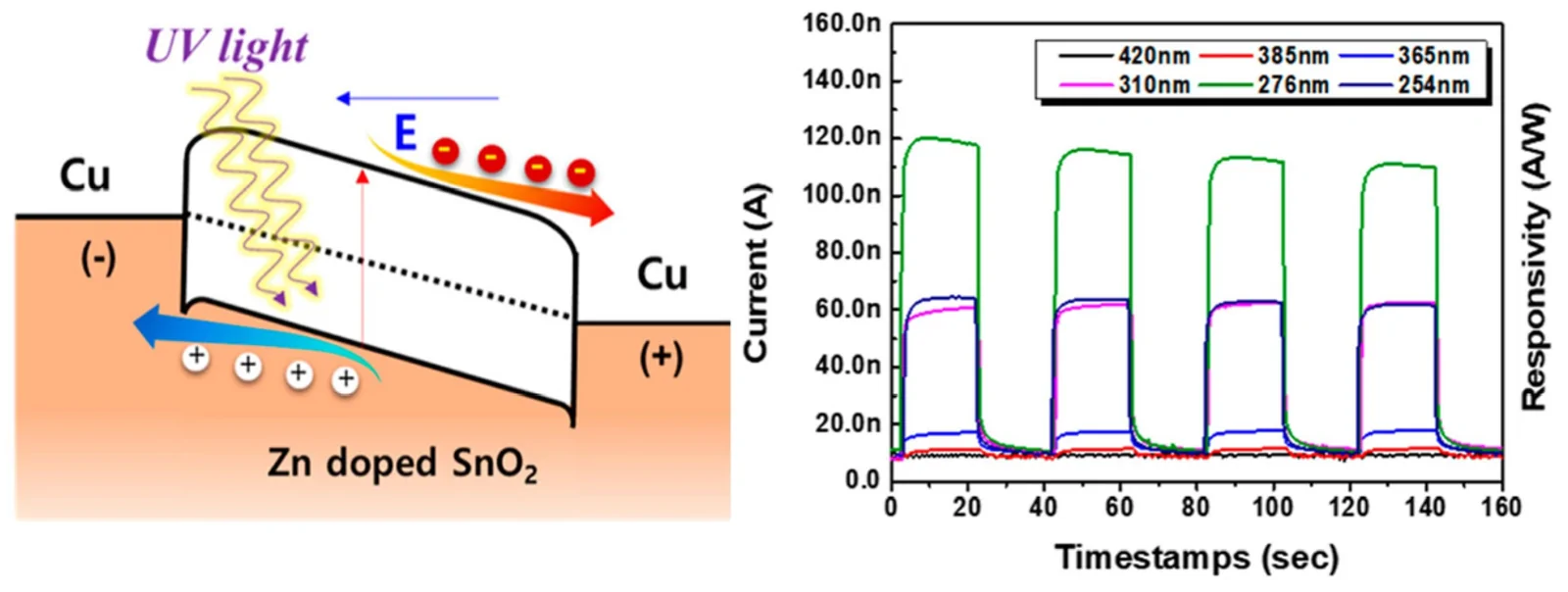
The band diagram and I-T curves under different wavelengths of illumination of the Zn-doped SnO_2_ TFT UV PD [53]. Reproduced with permission from Ref. [53]. Copyright © 2023 American Chemical Society.

Wide-bandgap oxides like SnO_2_ and IGZO are also used in SBUV detection research. SnO_2_ has a wide bandgap and good chemical stability, but its selectivity and response recovery in the DUV band are easily affected by oxygen vacancies, surface adsorption states, and deep-level defects. Lee et al. improved the response performance of SBUV detectors by controlling PPC in Zn-doped SnO_2_ thin films [53] (Figure 12), indicating the importance of modulating defect states and carrier relaxation processes. Chiu et al. reported a DUV-sensitive thin-film transistor using Ta_2_O_5_ as the gate dielectric and a-IGZO as the channel layer, achieving a mobility of 48.5 cm^2^/Vs in the dark and a photocurrent response exceeding four orders of magnitude under 254 nm [54].

Overall, the development of DUV and SBUV detection mainly relies on the spectral selectivity of wide-bandgap and ultra-wide-bandgap oxide semiconductors, while also being influenced by thin-film structure, defect states, and interface quality. The Ga_2_O_3_ system has received widespread attention due to the reasonable match of its absorption edge with the SBUV band. Crystalline Ga_2_O_3_ focuses more on high-quality channels and low dark current, while amorphous Ga_2_O_3_ has advantages in low-temperature preparation, large-area uniformity, and process compatibility. Materials like MgZnO, ZnGaO, ZnGa_2_O_4_, and SnO_2_ further expand the composition tuning and defect control ideas for solar-blind detectors. Subsequent research still needs to improve response recovery speed, environmental stability, bias stability, and array consistency, while maintaining DUV selectivity and low dark current, to advance MO TFT SBUV PDs towards imaging, communication, and integrated sensing applications.

#### 3.2.2. Near-Ultraviolet and Ultraviolet Detection

Near-ultraviolet and UV detection typically covers the approximately 280–400 nm band, finding applications in environmental monitoring, biological detection, UV curing, wearable sensing, and image perception. Since the absorption edges of many wide-bandgap metal oxide semiconductors lie in the near-ultraviolet region, near-ultraviolet and UV detection has become a relatively active research direction in MO TFT PDs.

**Figure 13:**
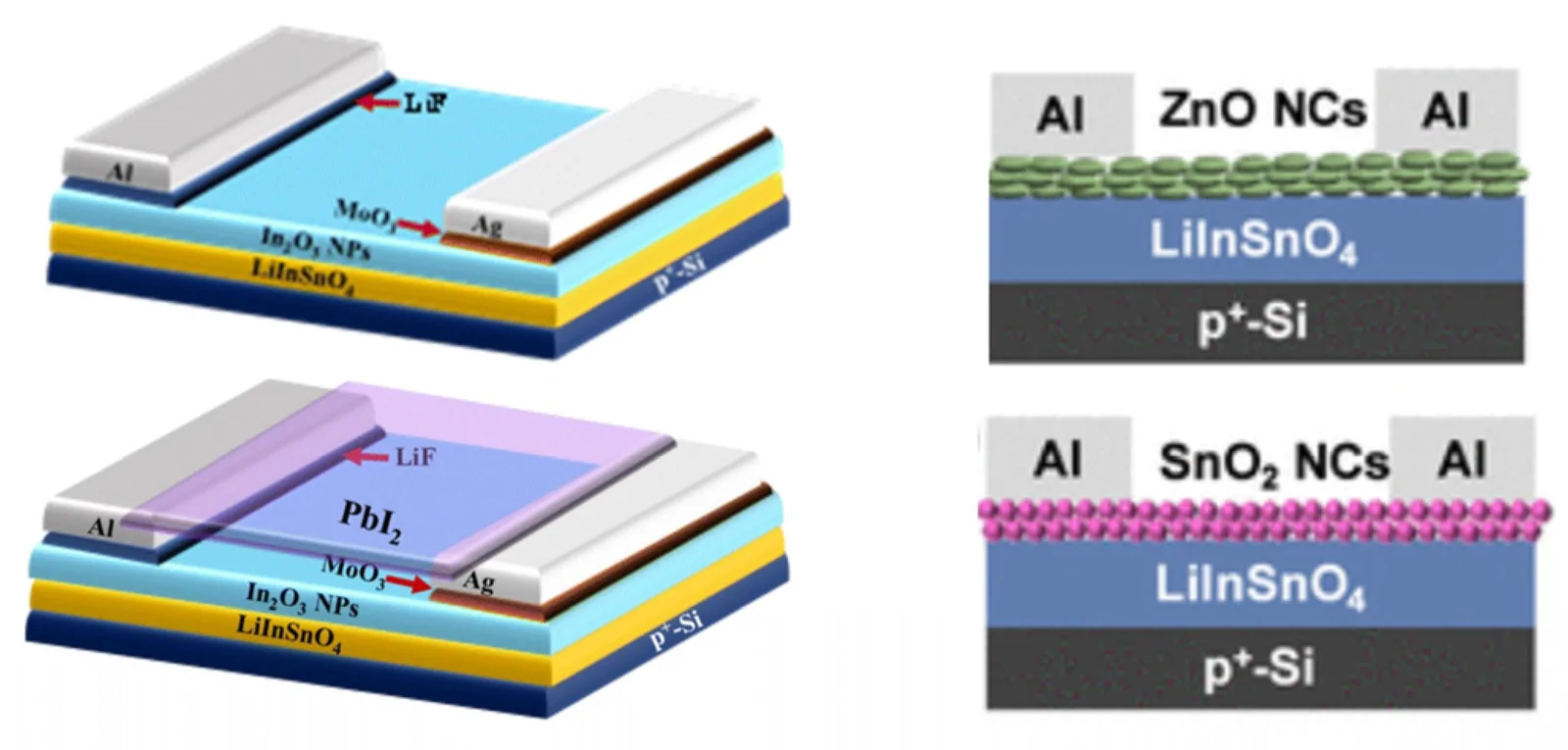
Schematic diagram of a UV PD TFT structure with PbI/In_2_O_3_ nanoparticles, and ZnO/SnO_2_ as the channel [56,57]. Reproduced from Ref. [56] under the Creative Commons Attribution-NonCommercial 3.0 International License (CC BY NC 3.0). Reproduced with permission from Ref. [57]. Copyright © 2026 American Chemical Society.

ZnO, SnO_2_, and In_2_O_3_ are common material systems used in UV PDs. ZnO has an absorption edge close to the UV region, SnO_2_ possesses good chemical stability and optical transparency, and In_2_O_3_ has high electron transport capability. Pal et al. fabricated a UV TFT PD using ZnO nanosheets, achieving a detectivity as high as 1.24 × 10^13^ Jones [55]. Subsequently, the same group prepared In_2_O_3_ nanoparticles and combined them with PbI to achieve visible-blind UV detection, with the response band tunable in the 395–445 nm range [56]. Later, they synthesized ZnO and SnO_2_ nanocrystals, and the fabricated TFTs exhibited a strong UV photoresponse [57] (Figure 13).

Near-ultraviolet and UV detection typically places more emphasis on dark current control, response recovery, and repeatability under cycling tests. Therefore, methods such as doping modulation [58], oxygen partial pressure optimization [59], and annealing treatment [60] are often used to regulate the film defect state and interface quality, thereby improving the UV response behavior of the devices.

**Figure 14:**
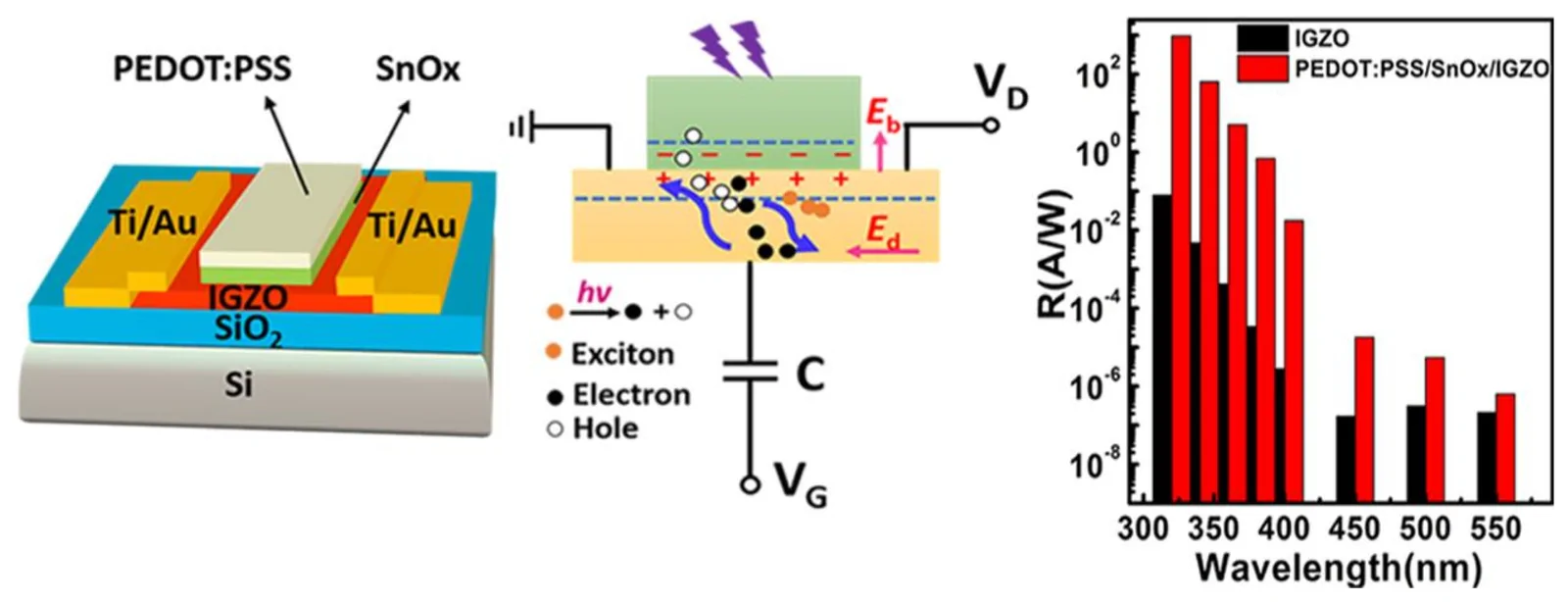
Schematic diagram, equivalent circuit diagrams and responsivity under illumination at different wavelengths of the PEDOT:PSS/SnOx/IGZO PD [64]. Reproduced with permission from Ref. [64]. Copyright © 2018 American Chemical Society.

IGZO and other amorphous oxide semiconductors hold an important position in near-ultraviolet and UV detection. Compared to crystalline oxides, IGZO stands out for low-temperature processing, large-area uniformity, and a relatively mature TFT process foundation, making it suitable as a channel material for UV photodetection. Under UV illumination, IGZO devices typically exhibit significant source-drain current modulation, but their response process is also easily affected by band tail states, oxygen vacancies, and interface traps. Related research often focuses on film oxygen content [61], annealing atmosphere [62], gate dielectric interface, and passivation layer design [63] to reduce current residue and Vth drift after illumination and to improve device stability under multiple illumination cycles. In addition to film and interface regulation, device structure design is also an important approach to enhancing the UV detection performance of IGZO-based devices. Yu et al. designed a hybrid-structure visible-blind UV photodetector based on an IGZO thin-film transistor coupled with a PEDOT:PSS/SnO_x_/IGZO p-n heterojunction, achieving a high photoresponsivity of 984 A/W, a UV-visible rejection ratio of 3.5 × 10^7^, and a specific detectivity of 3.3 × 10^14^ Jones [64] (Figure 14). Chen et al. fabricated short-channel IGZO dual-gate TFTs and found that short-channel devices exhibited a stronger UV photoresponse compared to long-channel devices [65].

Near-ultraviolet and UV detection provide a relatively straightforward photoresponse scenario for MO TFT. Devices can achieve a significant photoresponse through material composition, process conditions, and interface regulation, but their performance is still limited by defect-related relaxation processes. As detection requirements further extend to the visible light region, relying solely on the intrinsic absorption of wide-bandgap oxides will gradually become insufficient, necessitating the introduction of new spectral extension strategies.

#### 3.2.3. Visible Light Detection

As the detection band extends from the ultraviolet region to the visible light region, the core issue faced by MO TFTs gradually shifts from intrinsic light absorption to the construction of a response to low-energy photons. Most metal oxide semiconductors have relatively wide bandgaps, with absorption edges typically located in the UV or near-UV region. Therefore, a single wide-bandgap channel has weak direct absorption of visible light. For visible light detection, device design needs to introduce physical processes or functional structures capable of responding to low-energy photons while maintaining the TFT’s low dark current, good switching characteristics, and process compatibility. Consequently, visible light detection no longer primarily relies on the intrinsic band-edge absorption of the material but emphasizes the synergistic effect of mechanisms such as sub-bandgap states, composite channels, and interface charge transfer.

**Figure 15:**
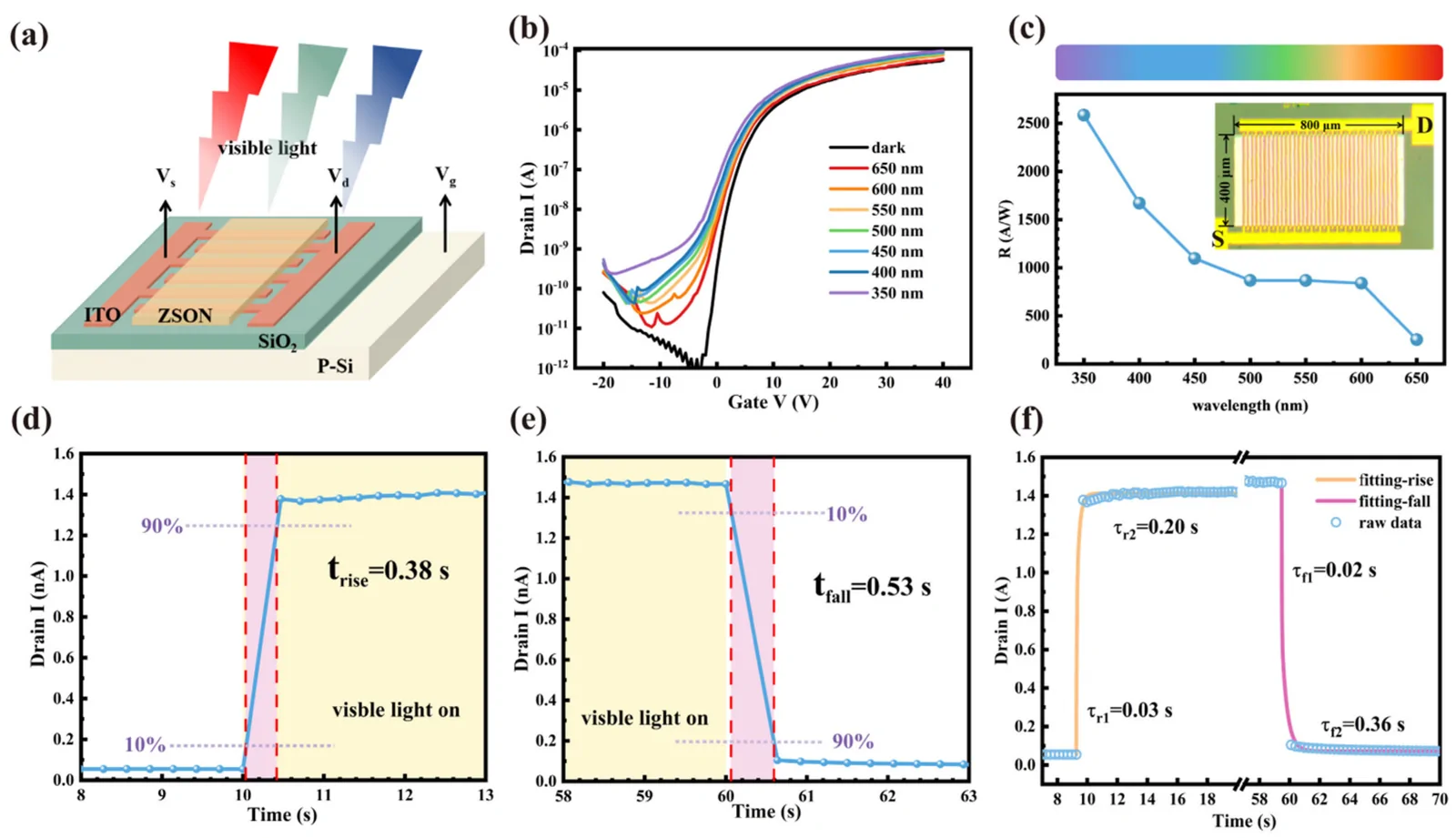
(**a**) Schematic of ZSON TFT PD structure. (**b**) Transfer curves. (**c**) Light responsivity of ZSON PD under different wavelengths of light illumination and in the dark. The inset shows a top-view of the device microscope; time-resolution photocurrent images of the PD under 450 nmillumination of (**d**) current-rise and (**e**) current-fall phases. (**f**) Experimental and fit curves of the rise and fall processes [67]. Reproduced with permission from Ref. [67]. Copyright © 2024 IEEE.

Defect-state engineering is a common approach to extend the visible light response of MO TFTs. V_O_, band tail states, and deep-level defects can form sub-bandgap absorption channels within the bandgap, allowing visible light to induce carrier generation or defect charge state transformation under certain conditions, further causing channel conductance changes. Xie et al. effectively narrowed the bandgap of ZnSnO to the visible region (~1.6 eV) by introducing N, enabling ZnSnON TFTs to achieve a high photoresponsivity exceeding 6 × 10^3^ A/W in the 400–800 nm range [66]. Based on this, subsequent work further optimized the N content and introduced metal-rich conditions, shortening the response time to 0.38/0.53 s while maintaining the visible light response advantage brought by N, compensating for the response lag caused by N-related defects [67] (Figure 15). Tarsoly et al. proposed another gradient annealing method, constructing a bilayer amorphous IGZO thin film structure with high-temperature annealing at the bottom and no annealing on top. Visible light response was achieved by utilizing defect states in the top layer to trap photogenerated charges [68]. Chen et al. further confirmed this mechanism from the perspective of material intrinsic defects, systematically comparing the photoelectric responses of five oxide TFTs including IGZO, IZO, and In_2_O_3_. They found that IZO and In_2_O_3_ devices with higher V_O_ content exhibited higher photocurrent under 450 nm visible light [69]. These methods do not require the introduction of additional complex photosensitive layers and have certain process simplicity, but their limitations are also evident. Excessive defects may lead to increased dark current, Vth drift, and enhanced PPC, reducing device recovery speed and repeatability stability.

Another approach is to enhance visible light absorption through composite channels or photosensitive functional layers. For wide-bandgap oxide TFTs, photosensitive components such as narrow-bandgap oxides [70,71], quantum dots [72], perovskites [73], organic semiconductors [74], or two-dimensional materials [75] can be introduced, so that the absorption of low-energy visible light mainly occurs in the composite channel or additional functional layer, followed by electrical readout through interface charge transfer, photo-induced doping, or channel conductance modulation.

**Figure 16:**
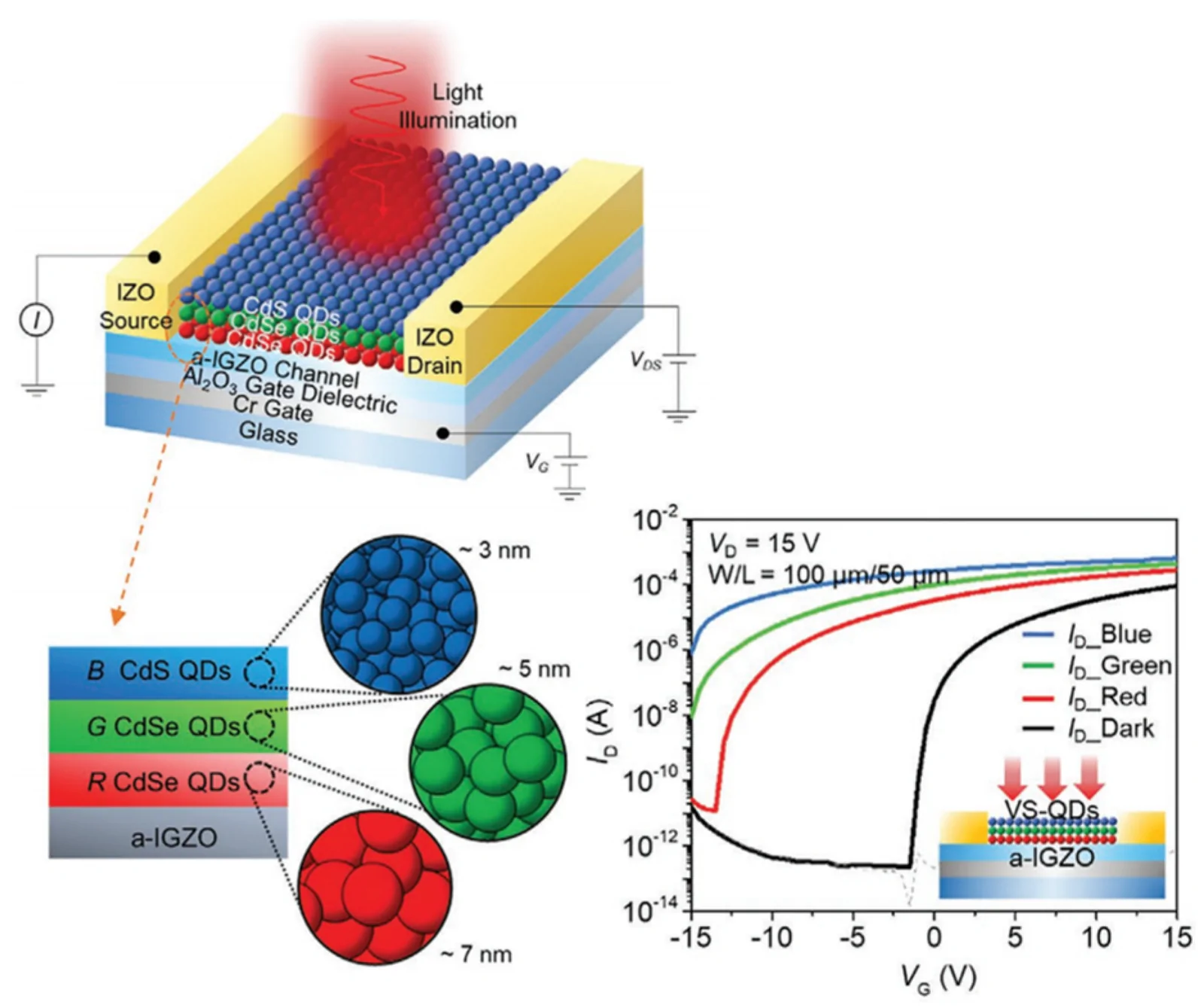
Schematic 3D view and optoelectronic characteristics of the VS-QD/a-IGZO PD [78]. Reproduced with permission from Ref. [78]. Copyright © 2021 John Wiley and Sons.

Jang et al. successfully achieved a response to red light (635 nm) by constructing an in situ IGZO/ITON heterojunction phototransistor [76] (Figure 16). Cho et al. coated CdSe/ZnS quantum dots on the ZnO surface, achieving a strong response to visible light, while the photocurrent in the near-infrared band was significantly lower [77]. Furthermore, Kim et al. achieved full-color visible light response by compositing quantum dots with IGZO [78] (Figure 16) Chen composited small organic molecules (C_70_/DBP) with IGZO transistors, obtaining a fast response of approximately 50 ms and a sensitivity exceeding 10^4^ under 625 nm visible light, significantly enhancing the device’s visible light detection performance [79].

The composite material strategy can usually more effectively enhance the visible light response and provide more room for band adjustment and sensitivity optimization. However, composite structures also introduce new interface defects, band alignment issues, and environmental stability concerns. Some photosensitive materials may also increase process complexity and affect large-area fabrication uniformity.

Visible light detection is an important transitional step for MO TFT PDs moving from intrinsic UV response to broadband spectral perception. Defect-state engineering, composite channel design, and photosensitive layer modification provide different spectral extension paths, but they also correspond to issues such as dark current control, response recovery, interface stability, and process compatibility. Subsequent research needs to further improve the absorption efficiency of low-energy light and the efficiency of interface charge transfer, while reducing current residue and device drift after illumination, to enhance the stability and reliability of MO TFTs in visible light detection. Compared with UV detection, visible light detection relies more on material combinations and interface design, and also provides a foundation for further expansion to NIR and broadband detection.

#### 3.2.4. Near-Infrared and Broadband Detection

When the detection band further extends to the NIR region, the issue of low-energy light response in MO TFTs becomes more prominent. The intrinsic absorption of most oxide semiconductors struggles to cover the NIR band. Therefore, NIR detection generally cannot rely solely on the band-edge absorption or defect state response of the channel itself, but requires the introduction of functional materials with strong low-energy light absorption capabilities.

PbS quantum dots have tunable narrow bandgaps and strong NIR absorption ability. When combined with an IGZO channel, the photosensitive layer absorbs NIR light and transfers carriers to the oxide channel, enabling NIR detection in the 700–1400 nm range [80]. Subsequent research further improved the charge transfer and device stability between PbS quantum dots and IGZO through interface engineering methods such as Al_2_O_3_ doping and ligand modulation [81,82,83]. This indicates that NIR response depends not only on the absorption capacity of the photosensitive material itself but also on interface energy level alignment, carrier transport, and charge retention processes. Compared with visible light detection, NIR photons have lower energy, imposing higher requirements on the absorption capacity of the photosensitive component, interface charge transfer efficiency, and carrier collection process. Device performance is also easily affected by interface energy level alignment, photosensitive layer stability, and charge retention.

**Figure 17:**
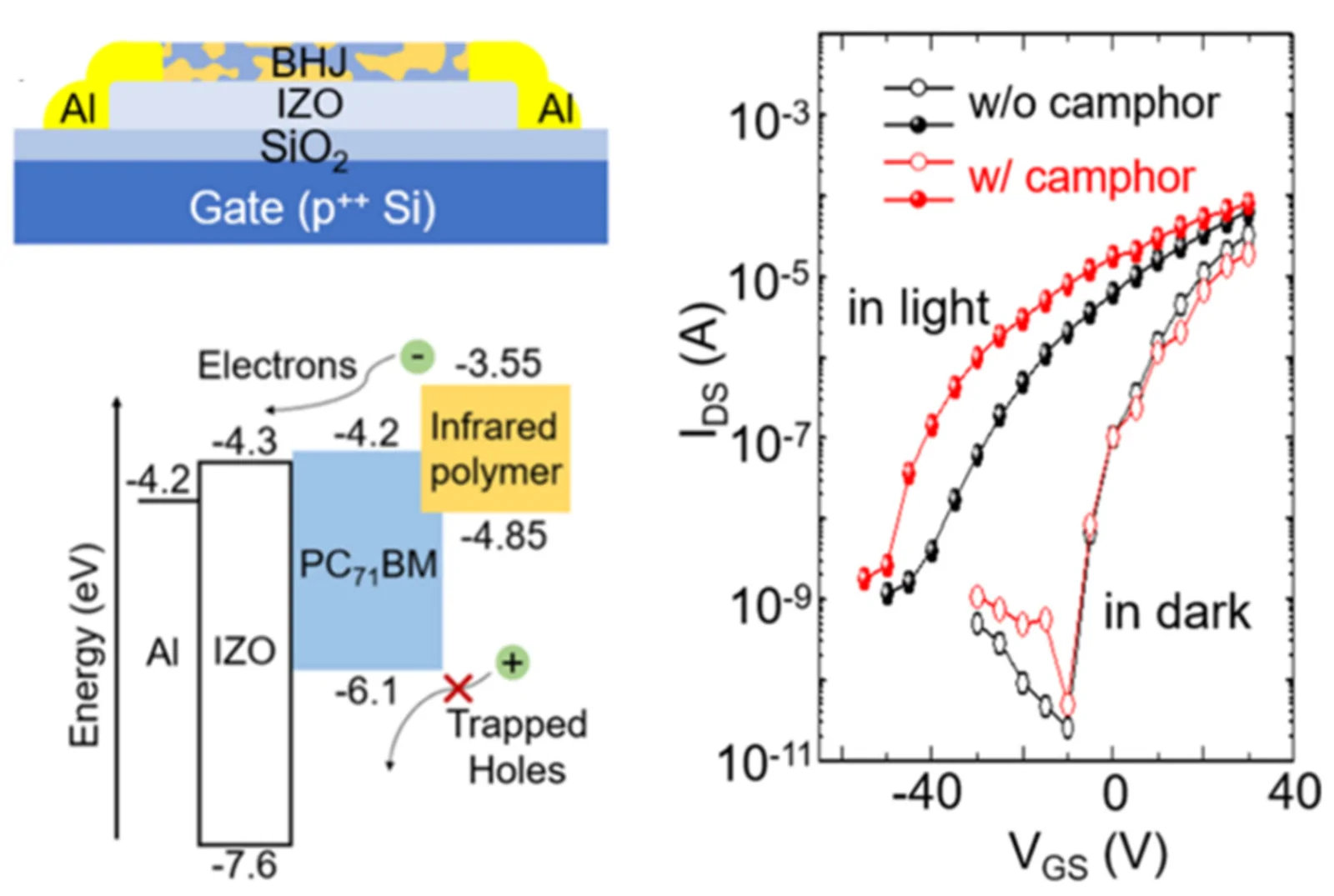
Structural diagram of the IZO/BHJ PD. Energy diagram illustrating electron transfer from the BHJ to IZO, whereas holes are trapped in BHJ. Transfer characteristics of the PDs. The light was from an LED emitting at a 940 nm wavelength with a power of 50 mW/cm^2^ [86]. Reproduced with permission from Ref. [86] Copyright © 2019 American Chemical Society.

The focus of broadband detection differs somewhat from that of NIR detection. NIR detection is primarily concerned with establishing a response to low-energy light, while broadband detection emphasizes continuous response and coordinated readout across multiple wavelength bands, including UV, visible, and NIR. To achieve broadband detection, devices typically need to incorporate multiple absorption channels simultaneously, so that optical signals of different energies can be converted into current output on the same TFT platform. For example, through the energy level alignment and field-effect modulation of organic/inorganic semiconductors, IGZO/C8-BTBT heterojunction phototransistors achieved broadband detection from UV to NIR with a high detectivity [84]. Similarly, by compositing with an organic photosensitive layer, the response spectrum of the device can be further extended to 450–950 nm [85]. Kim et al. constructed an organic and IZO bilayer TFT structure, expanding the response band range to 500–1400 nm; it exhibited performance superior to commercial germanium photodiodes in the spectral range below 1300 nm [86] (Figure 17). However, the absorption positions, carrier generation methods, and charge transport paths corresponding to different bands may not be identical, easily leading to variations in responsivity, response speed, and recovery processes with wavelength. Therefore, broadband detection not only requires expanding the absorption range but also paying attention to the consistency and stability of the response across bands.

#### 3.2.5. Multi-Band Photodetection and Spectral Selectivity

The aforementioned photodetection in different bands primarily focuses on whether the device can obtain an effective response within a specific wavelength range or expand the response range to a broader spectrum. However, in actual light environments, incident light typically consists of multiple bands combined. Obtaining a strong or wide photoresponse alone may not necessarily meet application requirements. For applications such as imaging sensing, environmental monitoring, and intelligent vision, devices also need to produce distinguishable outputs for optical signals of different wavelengths. Therefore, research on MO TFT PDs is gradually advancing from single-band response and broadband coverage to high spectral selectivity and multi-band distinguishable readout.

Broadband detection primarily addresses the issue of expanding the response range, while spectral selective detection further focuses on the degree of differentiation and readout reliability among optical signals of different wavelengths. For MO TFTs, this differentiation capability can come from differences in material intrinsic absorption edges, the band selectivity of composite channels or photosensitive layers, or can be achieved by changing the output characteristics at different wavelengths through defect state distribution, interface charge transfer, and bias condition modulation [78,87,88]. Multi-band detection emphasizes the synergy and separation between different response channels. If the response processes of multiple bands are highly overlapping, although the device can achieve broadband response, it is difficult to accurately determine the wavelength range of the incident light [89].

Therefore, the design of multi-band TFT photodetectors requires a balance between response range, band differentiation, and readout stability. Response overlap between different absorption channels, interface charge retention, and defect trapping may lead to band crosstalk, response delay, and signal drift, thereby reducing the reliability of multi-band identification. Subsequent research needs to further optimize material absorption, interface quality, and device readout methods, so that MO TFTs can not only respond to multiple bands but also achieve more stable and distinguishable spectral information readout.

### 3.3. Summary of This Chapter

This chapter discussed the research progress on MO TFT PDs. It first introduced the basic concepts and common performance indicators of PDs, then discussed the development characteristics of DUV and SBUV detection, near-UV and UV detection, visible light detection, NIR and broadband detection, and multi-band photodetection according to the response band. Table 1 summarizes the key parameters. In general, the development of MO TFT PDs can be understood as a process gradually moving from the intrinsic UV response of wide-bandgap oxides to low-energy light response, broadband coverage, and multi-band distinguishable response.

In DUV and SBUV detection, the material bandgap and absorption edge position determine the device’s spectral selectivity. In near-UV and UV detection, the devices must account for the relationship between intrinsic photoresponse and defect relaxation. When the detection band extends to the visible and NIR regions, insufficient low-energy light absorption becomes the main limitation, making composite channels, photosensitive functional layers, and interface charge transfer important control methods. Further toward broadband and multi-band detection, the research focus shifts from simply expanding the response range to improving the differentiation and response stability of signals from different bands.

From the perspective of device performance control, the performance improvement is usually not determined by a single factor. Wide-bandgap oxides are beneficial for reducing the dark current background and obtaining UV-selective response, but their intrinsic absorption in the visible and NIR regions is limited. Defect states and interface processes can enhance the photoresponse but may also cause PPC, recovery delay, and Vth drift. Composite channels and photosensitive layer modification can expand the response band but impose higher requirements on interface stability and process compatibility. Therefore, optimization requires a balance between responsivity, dark current, response speed, spectral selectivity, and long-term stability.

From a comparative perspective, the differences among various materials and structural approaches are not limited to the response wavelength range, but are more evident in the trade-offs between responsivity, dark current, response speed, and spectral selectivity. Ultra-wide bandgap oxides are better suited for UV detection in the solar-blind range, offering the advantages of low background response and high spectral selectivity, but with limited responsivity to low-energy light; mature oxide TFT systems, such as IGZO, are better suited for array fabrication and active-matrix readout, but their response to visible and near-infrared light typically relies on defects or composite structures; coupling photosensitive layers such as quantum dots and organic semiconductors can effectively enhance the response to visible and near-infrared light, but this approach is also more prone to introducing issues such as interfacial hysteresis, environmental stability, and large-area uniformity. In contrast, all-oxide heterojunctions offer advantages in terms of process compatibility and stability, but their spectral extension capabilities are generally inferior to those of hybrid photosensitive layer structures. Therefore, when evaluating different detection approaches, one should not merely compare the highest responsivity; dark current, recovery speed, residual PPC, environmental stability, and array compatibility must also be considered simultaneously.

As research progresses from single-band response to broadband and multi-band distinguishable response, MO TFT PDs still have further room for development in directions such as imaging sensing, environmental monitoring, and intelligent vision.

## 4. MO TFT Optoelectronic Synaptic Devices Research

The previous chapter discussed MO TFT photodetectors, focusing on the conversion of optical signals to electrical signals in different bands and their performance control. For PDs, the device typically pays more attention to indicators such as responsivity, dark current, detectivity, response speed, and stability. In OES devices, the conductance change induced by illumination is no longer just a transient detection signal but can be further transformed into a time-dependent state evolution to simulate plasticity behavior in biological synapses.

Therefore, this chapter starts from the functional requirements of OES and discusses the application of MO TFTs in light perception, weight modulation, and electrical characterization. It first introduces the basic concepts and key performance indicators of MO TFT OESs, then discusses related research according to the functional implementation path and further analyzes their development status in brain-inspired vision and neuromorphic computing.

### 4.1. Overview of MO TFT OESs and Key Performance Indicators

OES devices are a class of artificial synaptic devices that can modulate the device conductance state under the action of optical stimulation and represent synaptic weight changes through electrical signals. They are mainly used to simulate synaptic responses and plasticity changes induced by optical stimulation in biological visual systems. Unlike photodetectors, which primarily focus on the conversion of optical signals to electrical signals, optoelectronic synaptic devices emphasize the tunability and retention characteristics of the device conductance state after optical stimulation. For MO TFTs, processes such as photogenerated carriers, defect trapping/detrapping, interface charge accumulation, and gate voltage modulation can all participate in the light-induced conductance change, allowing illumination not only to produce an instantaneous current response but also to modulate the device state over a certain time scale.

The research focus for MO TFT OESs is not simply obtaining a large photocurrent, but rather how to convert the light-induced conductance change into a retainable, adjustable, and repeatedly updatable synaptic weight. The transient current response caused by illumination can correspond to the initial synaptic response under optical stimulation, while the slow current decay and state retention after illumination provide the basis for short-term memory, long-term memory, and weight modulation. Therefore, optoelectronic synaptic devices pay more attention to the evolution of photoresponse over time and stimulus history, rather than the current amplitude under a single illumination condition.

This functional orientation also makes the role of PPC different in OESs compared to PDs. In PDs, excessive PPC typically causes signal residue and recovery delay; in OESs, moderate charge retention and slow relaxation can be used to simulate memory retention and synaptic weight updates. Therefore, the control of PPC should not simply aim for suppression or enhancement but should be combined with synaptic functional requirements to control its time scale, reversibility, and cycling stability.

Based on the above characteristics, the performance evaluation of MO TFT OESs needs to shift from photoresponse intensity to synaptic functional integrity. In addition to the current change induced by light, attention should also be paid to whether the device conductance state can be continuously modulated, whether the optical stimulation history can be retained, whether the state update is reversible, and the repeatability under multiple stimulation and reset processes. Based on this evaluation logic, the following sections further introduce the key performance indicators commonly used in optoelectronic synaptic devices.

#### 4.1.1. Basic Synaptic Response and Short-Term Plasticity

Synaptic plasticity is the core function of OESs, typically used to describe the ability of the device conductance state to undergo tunable changes under external stimulation. Depending on the retention time and degree of modulation, synaptic plasticity can be divided into short-term plasticity and long-term plasticity [69]. Short-term plasticity (STP) and long-term plasticity (LTP) describe the response and regulatory mechanisms of neuronal synapses to stimuli on different time scales [70]. Specifically, short-term plasticity refers to rapid changes in synaptic weight that occur over a short period, closely following neuronal activity, but with a relatively short duration, typically ranging from milliseconds to minutes. Long-term plasticity refers to persistent changes in synaptic weight over a longer time scale, which can last from minutes to hours, or even longer.

Excitatory postsynaptic current (EPSC) is the most basic response form in optoelectronic synaptic devices. Under a single light pulse, the device source-drain current typically rises rapidly and then gradually decays after the illumination ceases. The current peak value reflects the device’s response intensity to a single optical stimulus, while the decay process reflects slow processes such as photogenerated carrier release, defect state trapping, and interface charge relaxation. Unlike PDs, which focus more on rapid recovery, OESs often need to utilize the moderate decay tail to simulate short-term memory and stimulus history retention. Therefore, EPSC is not only used to characterize the photoresponse intensity but also to analyze the device’s ability to transition from transient response to memory behavior [90].

**Figure 18:**
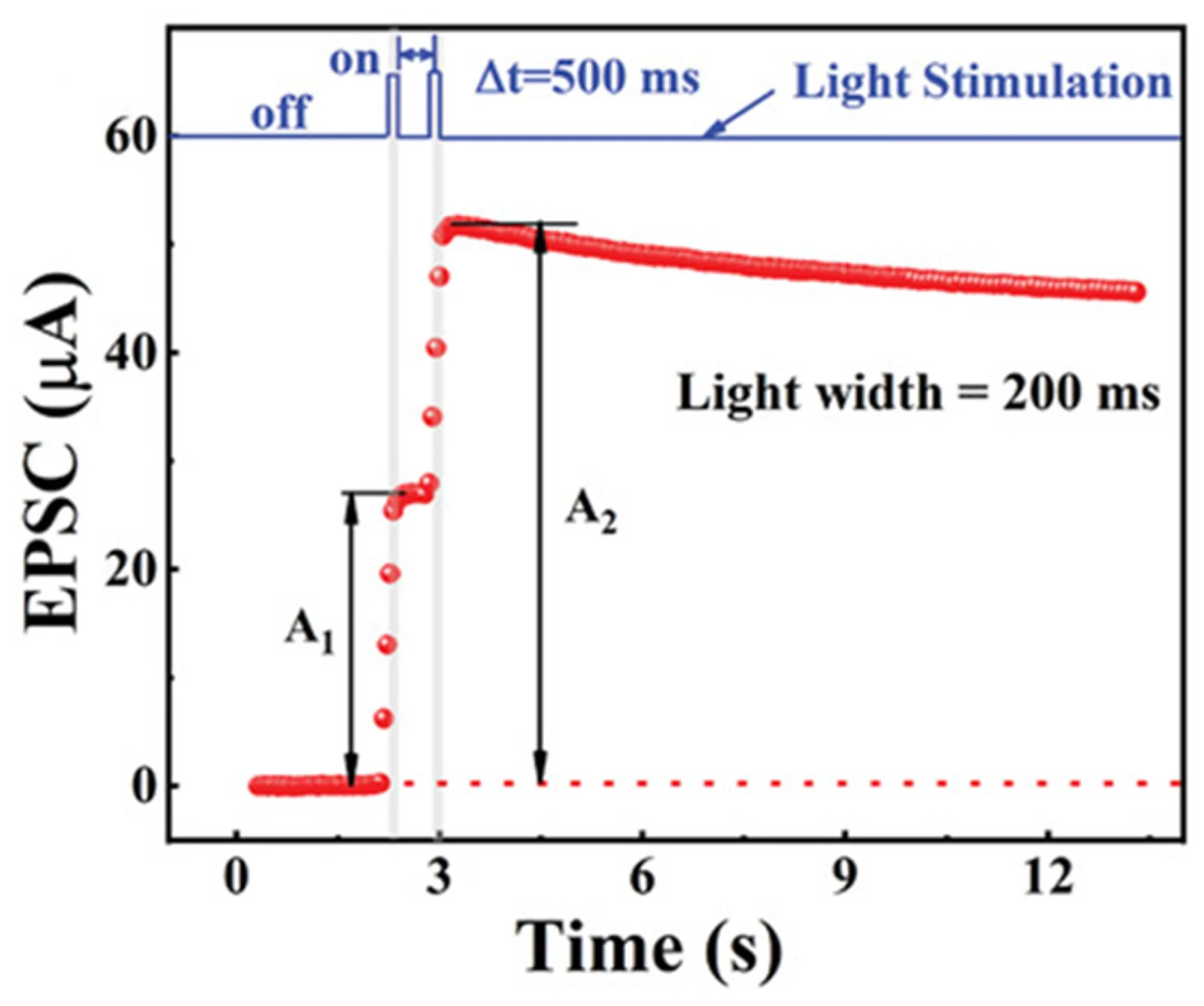
PPF behavior excited by optical pulse [92]. Reproduced with permission from Ref. [92]. Copyright © 2022 John Wiley and Sons.

Paired-pulse facilitation (PPF) is a typical manifestation of short-term plasticity, usually evaluated by applying two identical light pulses consecutively and comparing the response amplitudes of the two pulses. If the current response induced by the second pulse is higher than that of the first pulse, it indicates that the residual charge from the previous stimulation or the incompletely recovered conductance state has an enhancing effect on the subsequent stimulation (Figure 18). The PPF index is a metric used to quantify this enhancement effect, defined as

(12) $$PPF\;index=({EPSC}_{2}-{EPSC}_{1})/{EPSC}_{1}$$

where EPSC_1_ is the postsynaptic current generated by the device under the first pulse stimulation and EPSC_2_ is the postsynaptic current generated under the second pulse stimulation [91,92]. In biological synapses, real-time recognition of visual information is closely linked to the PPF behavior of postsynaptic neurons. Therefore, designing optoelectronic synaptic devices with a high PPF index is crucial for the development of artificial vision systems [93].

#### 4.1.2. Long-Term Weight Modulation and Application-Related Indicators

Under multi-pulse stimulation, the device conductance state may gradually increase or gradually decrease, corresponding to long-term potentiation (LTP) and long-term depression (LTD). Consecutive light pulses can cause accumulation of photogenerated carriers, trap occupancy, or interface charges, thereby gradually increasing the source-drain current or channel conductance and maintaining it for a certain period after stimulation ceases, i.e., LTP. Through reverse electrical stimulation, gate voltage pulses, or optoelectronic cooperative modulation, the device conductance can be gradually reduced, achieving weight inhibition, thus simulating LTD. For MO TFT OESs, long-term retention typically relies on defect-related slow recovery processes. Therefore, device structure design needs to strike a balance between retention time and erasability, and control the PPC intensity through back-channel passivation, heterojunction design, or composite channels to achieve more controllable long-term behavior [94,95,96]. In terms of application validation, many studies apply multi-level weights to neural network training or perception tasks and evaluate the contribution of device plasticity to system learning capability through recognition accuracy or noise immunity [94,97].

Spike-timing-dependent plasticity further reflects the device’s response ability to the order and time interval of stimuli. OES transistors can typically construct pre- and post-synaptic events through the relative time difference between light pulses and electrical gate pulses, causing the device to exhibit weight updates in different directions and magnitudes. The physical basis is that under different time sequences, the superposition modes of photogenerated carrier generation, electric field modulation, trap occupancy, and interface charge accumulation are different, leading to time-dependent changes in channel conductance. Related research indicates that three-terminal structures, due to their additional modulation terminal, are more likely to construct programmable timing learning windows, providing a device-level implementation path for sequence learning and event perception [7].

In addition to basic synaptic behavior, retention characteristics, forgetting behavior, and multi-level conductance modulation capability are also important aspects of evaluating OESs. Retention characteristics reflect the device’s ability to preserve the conductance state after optical stimulation ceases, while forgetting behavior describes the gradual decay of current or conductance over time [95,98]. For neuromorphic computing applications, devices also need to have continuous, repeatable multi-level conductance modulation capability to achieve fine-grained updates of synaptic weights. The conductance modulation range, update linearity, and symmetry of potentiation and depression processes affect weight mapping accuracy and system recognition performance, so the quality of a device cannot be judged solely based on the magnitude of a single photoresponse [99].

### 4.2. MO TFT OESs Categorized by Functional Implementation Path

The previous section introduced the basic functions and key performance indicators of MO TFT OESs. For such devices, the light-induced conductance change is usually related to processes such as channel defects, interface trapping, charge storage, and heterojunction or composite channel modulation. Different implementation paths affect the device’s responsivity, retention time, reversibility, and stability. Therefore, this section discusses related research according to the functional implementation path. By comparing the structural characteristics and control methods of different paths, the main development ideas of MO TFT OESs are further illustrated.

#### 4.2.1. Defect-Engineered Optoelectronic Synapses

Defect-engineered OESs mainly utilize intrinsic defects or artificially introduced defects in metal oxide semiconductors to achieve conductance modulation and memory behavior under optical stimulation. Metal oxide semiconductors commonly have V_O_, band tail states, deep-level defects, and surface adsorption-related states. These defects affect the generation, trapping, release, and recombination processes of photogenerated carriers, and further change the temporal evolution of channel conductance.

In PDs, such defects are often regarded as sources of response lag and stability degradation; in OESs, moderate defect trapping and slow release can be used to construct conductance states with memory characteristics. Shallow-level defects typically correspond to fast trapping/release processes, beneficial for forming short-term plasticity such as PPF and short-term memory. Deep-level defects have longer release times, easily causing PPC and longer-duration conductance retention, which can be used to simulate long-term memory and LTP behavior. Thus, defect energy level depth, defect density, and spatial distribution directly affect the device’s response amplitude, retention time, and recovery process.

**Figure 19:**
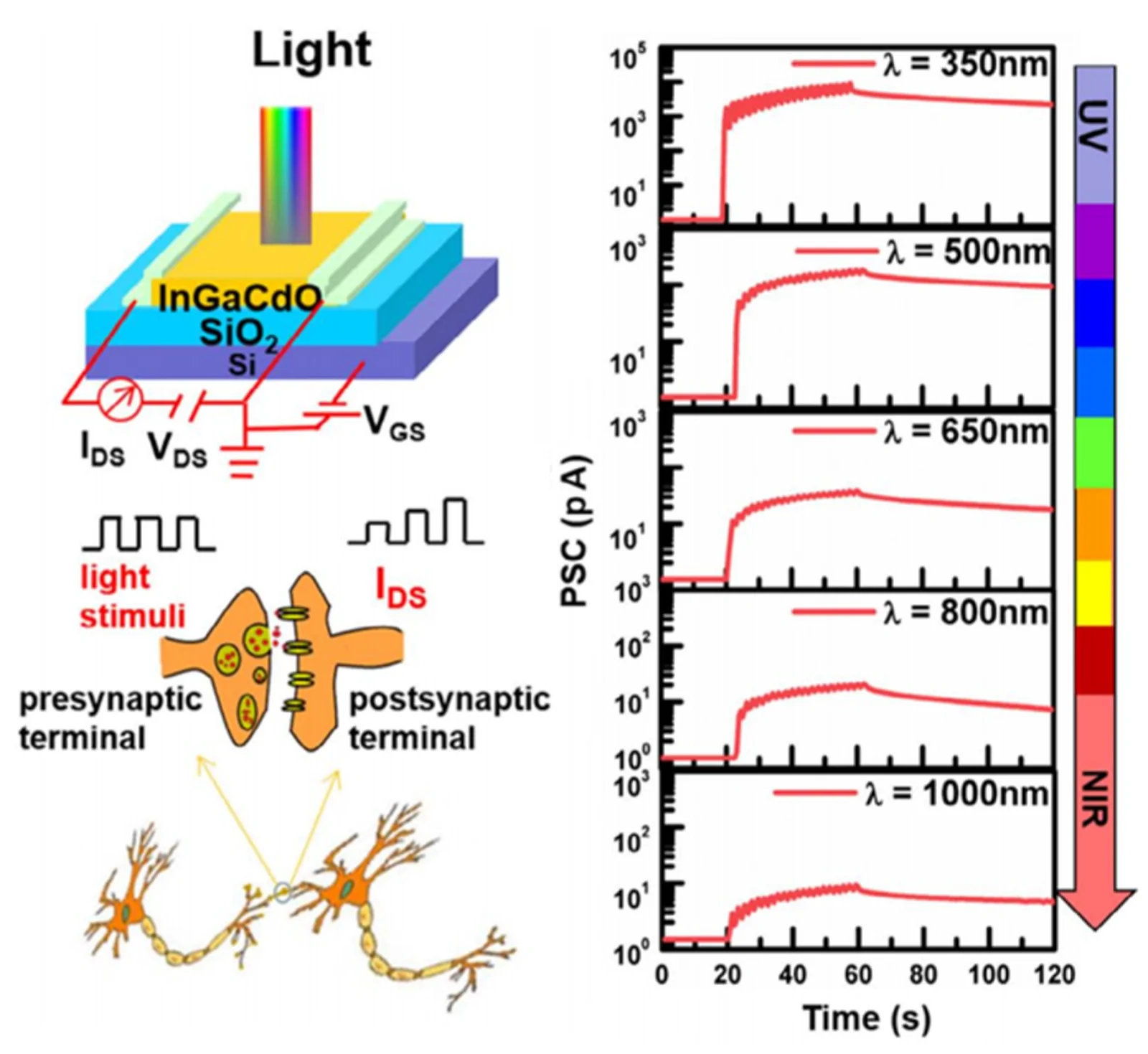
Device structure of IGCO TFT, synapse in the brain, and operation process of IGCO-based optoelectronic synapse device. PSCs triggered by different wavelengths ranging from UV to the NIR light with a constant pulse condition [9]. Reproduced with permission from Ref. [9] Copyright © 2020 John Wiley and Sons.

**Figure 20:**
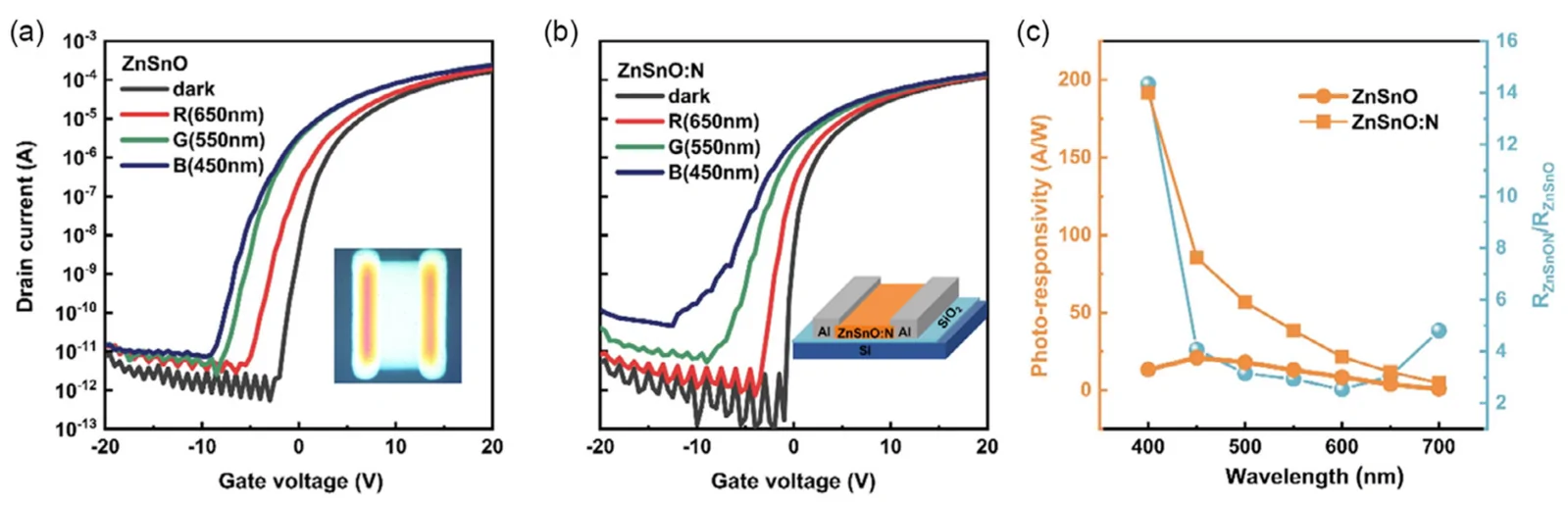
Photo-response properties of (**a**) ZnSnO and (**b**) ZnSnO:N TFTs under dark and Red/Green/Blue light; (**c**) comparison of the responsivity of ZnSnO and ZnSnO:N TFTs in visible light range [102]. Reproduced with permission from Ref. [102] Copyright © 2024 John Wiley and Sons.

Research on intrinsic defects and PPC behavior in oxide TFTs shows that defect engineering is not a single process step but involves comprehensive regulation of channel composition, oxygen vacancy formation, film densification, and surface state. Early studies showed that PPC in amorphous oxide semiconductors can be directly used to construct light-induced synaptic behavior, where the slow relaxation of conductance after illumination provides a physical basis for short-term memory, long-term memory, and weight modulation [100]. Subsequently, IGZO OES TFTs further demonstrated the application potential of MO TFTs in low-power optical stimulation response and selective attention functions [101]. In terms of material composition control, the IGCO OES TFT constructed by Duan et al. showed that the introduction of a Cd component enables high mobility, smaller bandgap, and larger oxygen vacancy recovery activation energy. The device exhibits light-induced synaptic response from UV to NIR, and its synaptic plasticity is closely related to the dynamic ionization and neutralization processes of oxygen vacancy-related defects [9] (Figure 19). Liu et al. modulated the ZnSnO channel through N doping, reducing the film bandgap from 3.04 eV to 2.90 eV and reducing non-radiative recombination centers, thereby enhancing visible light response and optoelectronic synaptic behavior [102] (Figure 20). These studies indicate that defect-engineered OESs should not simply be understood as increasing defect concentration, but rather requiring synergistic regulation of channel composition, defect energy levels, and carrier trapping/release processes to achieve a balance between photoresponse enhancement, state retention, and recoverability.

However, defect-engineered devices also have certain limitations. Since defect states simultaneously affect photoresponse and dark-state electrical characteristics, excessively strong defect-assisted response may lead to increased dark current, excessively slow response recovery, and decreased cycling stability. Non-uniformity of defect distribution may also cause device-to-device variations, affecting weight consistency in array applications.

#### 4.2.2. Interface-Trapping Optoelectronic Synapses

Interface-trapping OESs mainly utilize interface charge-trapping processes between the channel and adjacent dielectric layers, passivation layers, or the surface environment to achieve regulation and retention of the light-induced conductance state. Interface-trapping devices emphasize the influence of interface states, adsorption states, and interface electric fields on carrier transport and relaxation processes. For MO TFT, the interface quality between the channel and gate dielectric, the back-channel surface state, and external environmental adsorption can all alter the charge distribution after illumination, thereby affecting the device’s synaptic response.

Using the IGZO/Al_2_O_3_ structure as an example, light-induced synaptic behavior can be associated with charge-trapping processes at the IGZO/Al_2_O_3_ interface and within the Al_2_O_3_ dielectric layer, indicating that the gate dielectric interface not only affects TFT electrical performance but also participates in the formation of OES responses [103]. In electric-double-layer gated IGZO devices, interface ion accumulation and channel potential modulation further reduce the operating voltage and allow the synaptic behavior induced by optical stimulation to be modulated by the gate voltage [104,105]. Since the trapping and release times of interface traps typically have a distribution characteristic, the device conductance decay process often exhibits significant time dependence. Shallow interface traps facilitate faster reversible responses, while deeper interface traps can prolong conductance retention time but may also cause recovery delay and baseline drift.

**Figure 21:**
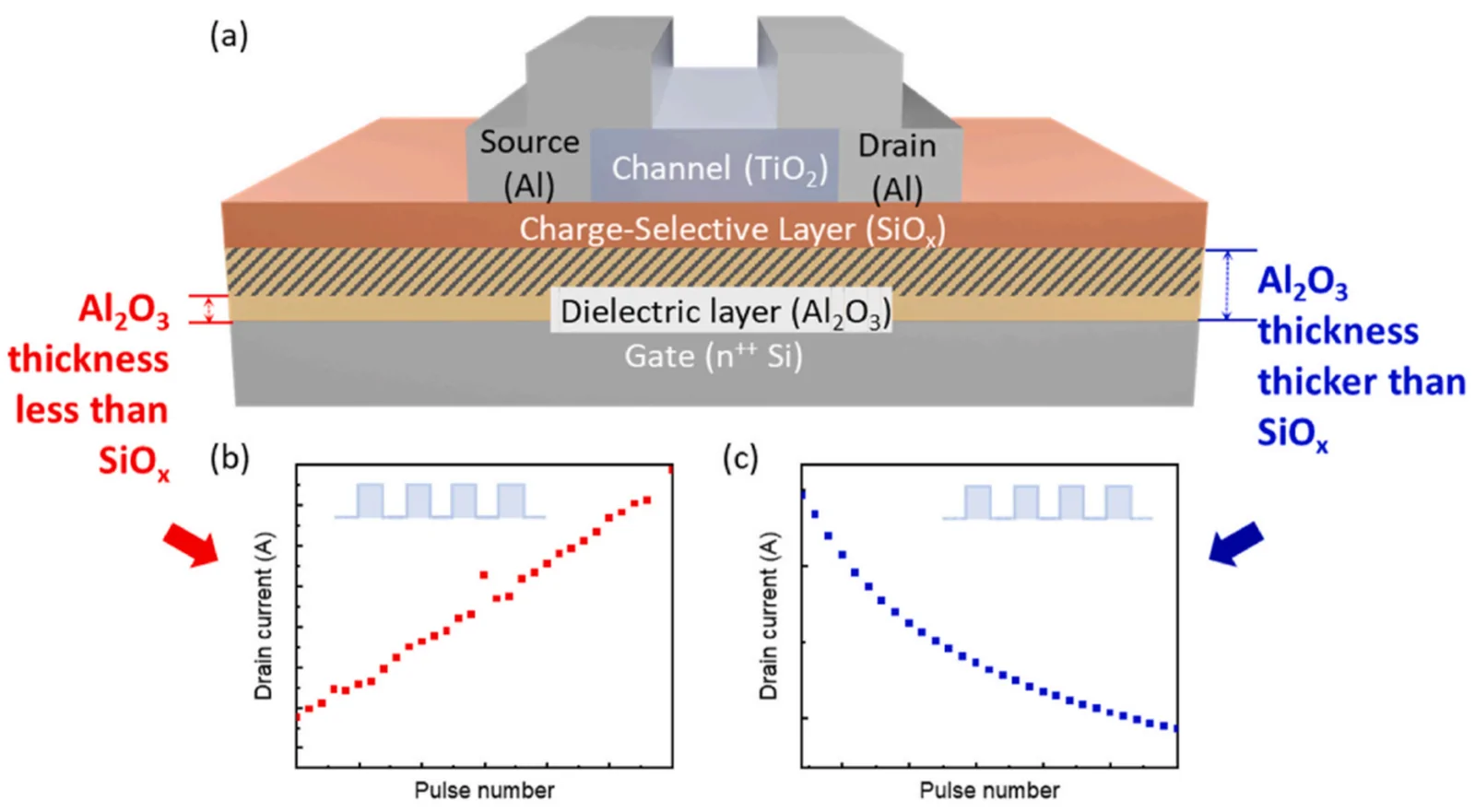
(**a**) Structure of the TiO_2_-based synaptic device featuring SiO_x_ and Al_2_O_3_ dual-gate dielectric layers. (**b**) The synaptic device with thinner Al_2_O_3_ layers exhibits potentiation behavior, while (**c**) the device with thicker Al_2_O_3_ layers demonstrates depression behavior [106]. Reproduced from Ref. [106] under the Creative Commons Attribution 4.0 International License (CC BY 4.0).

**Figure 22:**
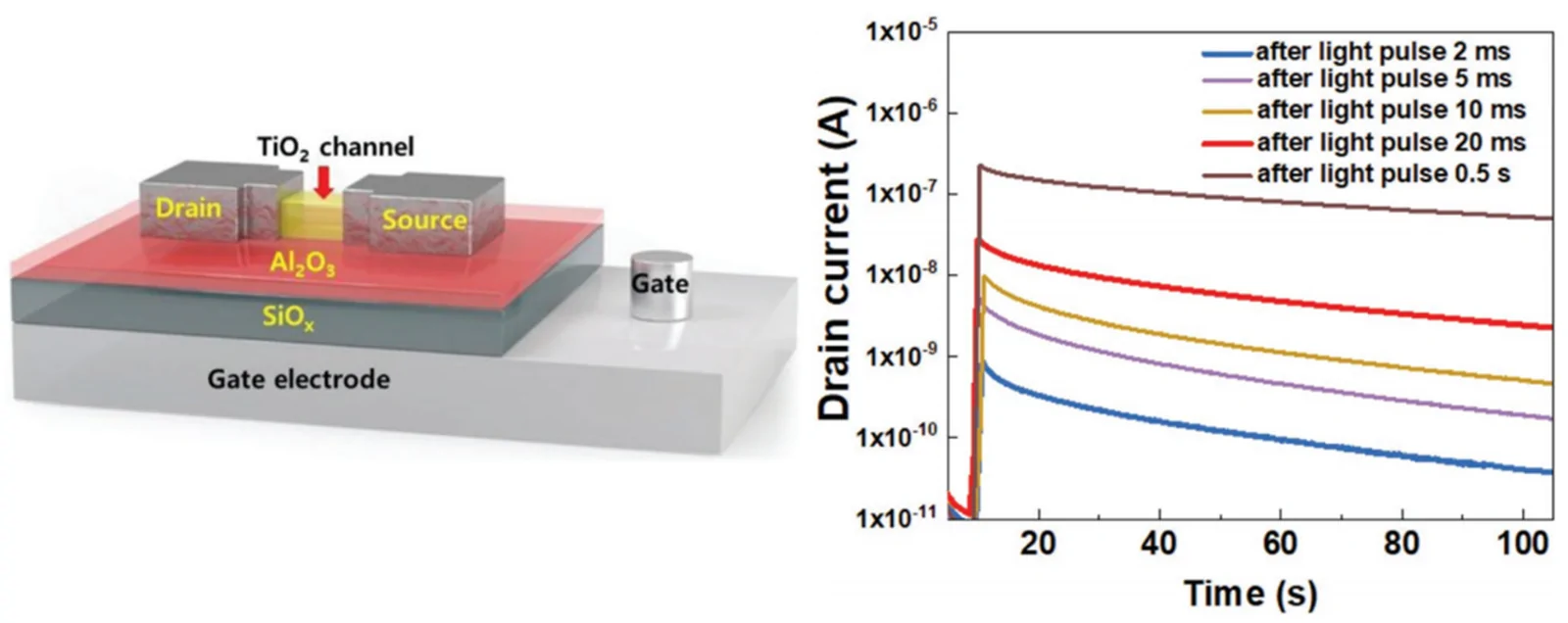
Structure of the synaptic transistor comprised a TiO_2_ channel layer, stacked Al_2_O_3_/SiO_x_ dielectric and EPSC curves at different applied pulse widths of light [107]. Reproduced with permission from Ref. [107] Copyright © 2022 John Wiley and Sons.

The interface-trapping process is closely related to the TFT structure and gate dielectric interface, where the gate dielectric material has an important influence on the interface electric field, trap distribution, and carrier trapping/release processes. Compared with traditional SiO_2_ dielectrics, high-k gate dielectrics such as Al_2_O_3_, HfO_2_, and ZrO_2_ can provide stronger gate control capability at lower operating voltages and enhance channel potential modulation through higher interface capacitance, which is beneficial for reducing device energy consumption and modulating conductance retention behavior after illumination [106] (Figure 21). Gate dielectrics and interface engineering can significantly affect the weight update characteristics of synaptic transistors. Lim et al. used Al_2_O_3_/SiO_2_ as the gate dielectric, enabling a TiO_2_ synaptic transistor to obtain a high number of states and good linearity and symmetry [107] (Figure 22). Jo et al. achieved highly linear IGZO synaptic devices through Al_2_O_3_-related interface-trapping process control [108]. Furthermore, V_O_, hydroxyl groups, or local defects in high-k dielectrics can also serve as charge-trapping centers, participating in the trapping and release processes of photogenerated carriers. Wang et al. constructed a solid-state electrolyte synaptic transistor, introducing Al_2_O_3_ as a charge-trapping layer, significantly enhancing the device’s long-term plasticity [109]. Thus, the gate dielectric is not merely an insulating layer in TFTs; it can also participate in synaptic behavior regulation through interface charge-trapping and release processes.

However, the memory effect brought by interface trapping is also easily accompanied by interface instability. Excessive or unevenly distributed interface traps may lead to baseline drift, Vth shift, and unstable cyclic response. Environmental adsorption and bias stress can further amplify such issues.

#### 4.2.3. Charge Storage and Ferroelectric Gate-Controlled OESs

In addition to channel defects and interface traps, introducing relatively independent state storage units in the TFT structure is also an important way to achieve OES functions. Charge storage devices typically introduce a charge-trapping layer or floating-gate unit in the gate dielectric system, allowing charges generated by optical or electrical stimulation to be stored near the dielectric layer and modulating the oxide channel potential through electrostatic coupling. The state storage location in such structures is relatively clear, which can reduce dependence on intrinsic channel defects to some extent, thus facilitating longer retention time and programmable conductance states.

A floating gate is a charge storage layer surrounded by insulating dielectrics and not directly connected to external electrodes. Under illumination or gate voltage pulses, carriers can be injected into the floating-gate or charge-trapping layer and maintained for a certain period after the stimulation is removed. The stored charge applies an internal gate voltage to the channel, causing continuous changes in Vth and source-drain current. By controlling light pulses, voltage pulses, and their combinations, the charge injection, trapping, and release processes can be regulated, enabling gradual updates of synaptic weights [110,111]. In such devices, the role of the intermediate layer should primarily be charge storage and its electrostatic modulation of the channel potential. If the device performance mainly originates from enhanced light absorption and interface charge transfer, the mechanism is closer to the heterojunction or composite channel type discussed later. Miao et al. reported a visible-light-driven IGZO OES transistor based on a CeO_x_ floating gate, achieving precise and reversible regulation of synaptic weights [112].

The advantages of charge storage OESs are strong retention characteristics, easy electrical modulation of the weight state, and facilitation of multi-level conductance updates [109]. However, the additional storage layer also increases device structure complexity and may introduce new interface defects and tunneling barriers. If charge storage is too strong, the device may suffer from difficult erasure and cyclic drift. If storage stability is insufficient, it is difficult to maintain long-term weight states. Therefore, such devices need to strike a balance between charge retention, reversible erasability, and operating voltage [109].

**Figure 23:**
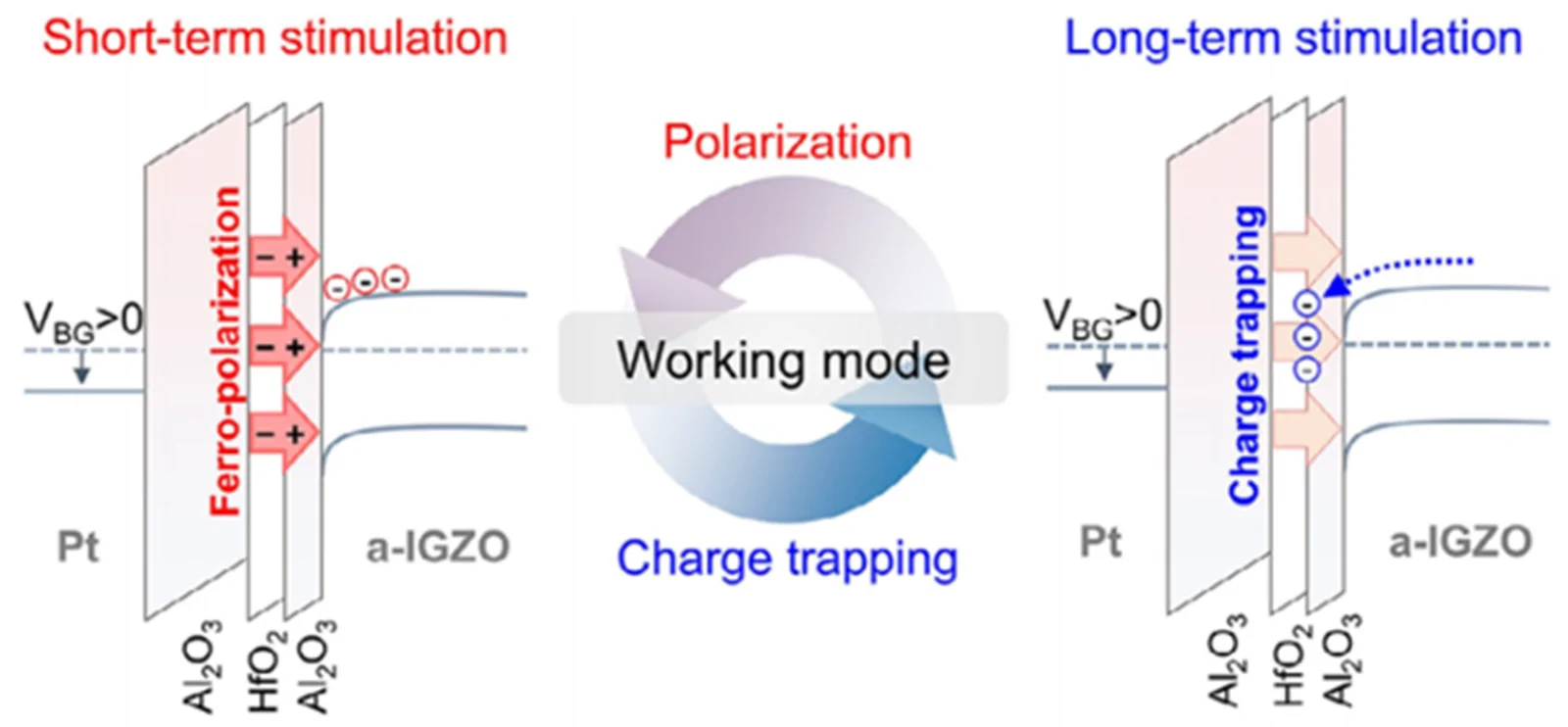
Schematic representation of the working mode transition between ferroelectric polarization and charge trapping [113]. Reproduced from Ref. [113] under the terms of the Creative Commons Attribution-NonCommercial-NoDerivatives 4.0 International License (CC BY-NC-ND 4.0).

Ferroelectric gate-controlled OES devices provide another state modulation approach. The core function of the ferroelectric gate dielectric lies in its switchable spontaneous polarization. An applied gate voltage can change the polarization direction and intensity of the ferroelectric layer, and the remanent polarization can still modulate the channel carrier distribution and threshold state after the voltage is removed. When a ferroelectric gate dielectric is combined with an oxide channel, photocarriers interact with the ferroelectric polarization field, interface charges, and channel potential, causing the device to exhibit conductance changes with both photoresponse and polarization memory characteristics. Kwon et al. used an Al_2_O_3_/HfO_2_ bilayer gate dielectric to induce a ferroelectric state, achieving dual-mode synaptic behavior combining ferroelectric polarization and charge trapping [113] (Figure 23). Recent research on IGZO/HZO ferroelectric field-effect transistors shows that after combining a Zr-doped HfO_2_ ferroelectric gate dielectric with an IGZO channel, the device can not only achieve synaptic weight modulation through electrical stimulation but also exhibit OES behavior under optical stimulation, improving the image recognition performance of neural networks through combined electrical and optical stimulation [114]. Further research on multispectral ferroelectric OES devices shows that the HZO polarization state can modulate the photocurrent decay rate and retention characteristics, allowing the device to exhibit tunable conductance responses under red-, green-, and blue-light illumination, and achieve transitions between short-term plasticity and long-term plasticity [115]. The advantage of ferroelectric gate-controlled devices is that their weight modulation has a relatively clear physical basis in polarization and does not completely rely on defect-trapping processes. By adjusting the ferroelectric polarization state through gate voltage pulses and then combining it with channel conductance changes induced by optical stimulation, functions such as optical writing, electrical modulation, or electrical reset can be achieved, providing a relatively stable state foundation for LTP, LTD, and multi-level weight modulation. Additionally, ferroelectric gate dielectrics are expected to enable strong electric field control at lower voltages, which helps reduce operating power consumption.

However, ferroelectric gate-controlled OES devices also face some issues. Polarization fatigue, leakage current, domain structure inhomogeneity in the ferroelectric layer, and interface defects with the oxide channel may affect the stability of weight updates and cyclic reliability. For thin-film TFT structures, it is also necessary to consider the low-temperature processing compatibility of ferroelectric materials, film uniformity, and interface quality with the oxide semiconductor. Therefore, the subsequent development of charge storage and ferroelectric gate-controlled devices needs to further optimize the storage layer energy level, dielectric layer thickness, ferroelectric polarization stability, and channel interface state, achieving a balance between long-term retention, reversible regulation, and process compatibility.

#### 4.2.4. Heterojunction and Composite Channel-Type OESs

Heterojunction and composite channel-type OESs mainly introduce functional materials with different band structures or light absorption characteristics into the MO TFT channel to change light absorption, carrier separation, and channel modulation processes. The focus of such devices is to utilize band differences and interface charge transfer between different materials, enabling photogenerated carriers to more effectively influence the oxide channel conductance. For wide-bandgap oxide TFTs, this approach is commonly used to enhance visible or NIR response and further modulate the conductance relaxation behavior after illumination.

**Figure 24:**
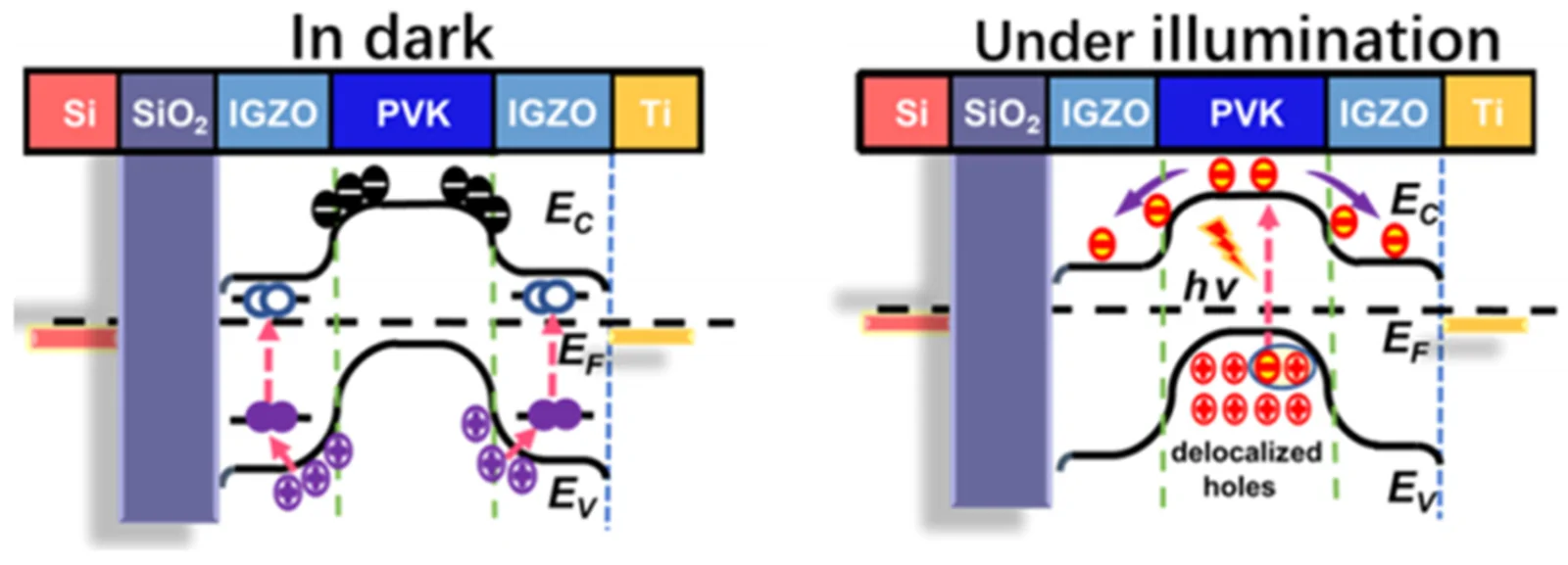
Schematic energy band diagram alignment of IGZO/PVK NPs/IGZO TFTs in dark and under illumination [117]. Reproduced with permission from Ref. [117] Copyright © 2021 American Chemical Society.

Heterojunction and composite channel-type devices can be discussed according to the material combination approach. One type involves introducing photosensitive components such as narrow-bandgap semiconductors, quantum dots, perovskites, organic semiconductors, or two-dimensional materials into oxide TFTs, utilizing their strong visible or NIR absorption ability to enhance photoresponse and achieve photogenerated carrier transfer to the oxide channel through band alignment [116]. Duan et al. constructed an IGZO/CsPbBr_3_ NPs/IGZO composite channel OES transistor. This device showed a synaptic gain 5–20 times higher than that of pure IGZO devices in the visible region, and could achieve complex synaptic behaviors such as short-term memory, long-term memory, and Pavlovian conditioning through modulation of light pulse frequency, light intensity, and gate voltage pulses [117] (Figure 24). Gao et al. reported an InP quantum dot-modulated ITZO phototransistor. After illumination, electrons generated in the InP QDs could spontaneously transfer to the ITZO channel, while holes remained in the quantum dots. The device exhibited photoresponse at both 550 nm and 650 nm—with responsivity in the long-wavelength region increased by about four orders of magnitude compared to pure ITZO devices—and achieved light adaptation, dark adaptation, transition from short-term memory to long-term memory, and 93% handwritten digit recognition accuracy [118]. However, the addition of quantum dots, perovskites, or other low-bandgap photosensitive materials may also bring issues related to environmental stability, interface compatibility, and large-area process consistency, which remain aspects requiring attention for the further application of this approach.

**Figure 25:**
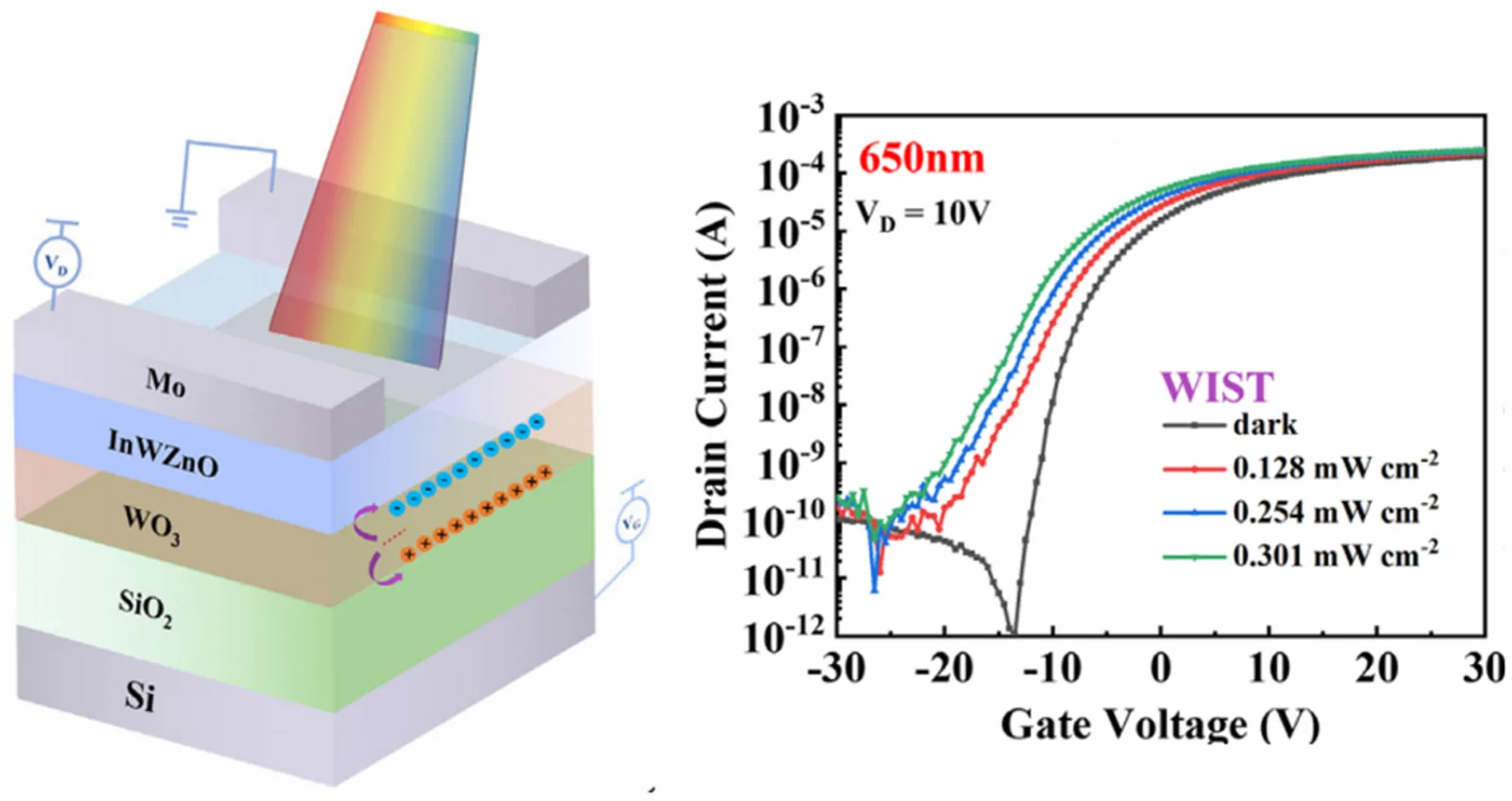
Structural diagram and transfer characteristics of WIST devices under 650 nm light illumination with intensity [95]. Reproduced from Ref. [95] under the terms of the Creative Commons Attribution-NonCommercial-NoDerivatives 4.0 International License (CC BY-NC-ND 4.0).

All-oxide heterojunctions and composite channels have gradually become an important direction for MO TFT OESs. This approach mainly utilizes differences in bandgap, carrier concentration, and defect distribution among different oxide semiconductors to regulate the separation, transport, and relaxation processes of photogenerated carriers. Chen et al. reported a WO_3_/InWZnO all-metal-oxide heterojunction OES transistor. This device exhibited OES response under 650 nm illumination and could achieve multi-level memory and artificial visual perception applications, indicating that all-oxide heterointerfaces can also be used to construct visible-light-responsive OES devices [95] (Figure 25). Furthermore, all-oxide heterojunctions or composite active layers such as In_2_O_3_/ZnO, ZnO/SnO_2_, and ITO/IGZO have also been used in optoelectronic artificial synapses, improving photoresponse intensity, memory retention, and recognition functions from perspectives such as heterointerface charge separation, oxygen vacancy regulation, and composite channel transport [96,119,120].

Heterojunction and composite channel-type devices provide effective ideas for low-energy photoresponse extension and conductance relaxation control in MO TFT OESs through material combination and band design. The external photosensitive material approach has greater advantages in enhancing visible and near-infrared absorption but needs to address material stability and interface consistency issues. The all-oxide composite channel approach is closer to traditional oxide TFT process platforms, with potential in terms of stability and array compatibility, but its low-energy light absorption capacity still needs further improvement. Therefore, subsequent research needs to strike a balance between light absorption enhancement, interface charge transfer, synaptic state retention, and process stability.

### 4.3. Summary: From Photodetection to Optoelectronic Synapses and Their Intelligent Vision Applications

This chapter discussed MO TFT OESs (Table 2). Compared with the photodetectors in Chapter 3, OESs are not merely designed to achieve efficient conversion of optical signals to electrical signals, but rather focus more on the temporal evolution, retention, and reversible regulation of the conductance state after optical stimulation. The two types of devices share physical processes such as light absorption, defect trapping/release, and interface charge modulation, but PPC has different functional implications in each: in photodetectors, it typically manifests as response lag; in OES devices, moderate charge retention can be used to simulate memory retention and synaptic weight updates.

Therefore, the key to MO TFT OESs is not simply enhancing the photoresponse, but regulating the formation, retention, and recovery processes of the light-induced conductance state through channel, interface, gate dielectric, and composite structure design. Different implementation paths ultimately point to the same goal: achieving a balance between responsivity, state controllability, cycling stability, and process compatibility, providing a stable device foundation for subsequent intelligent vision applications.

For OES devices, the key differences among various implementation approaches lie in the source of conductance state retention and its controllability. Defect modulation and interface trapping can relatively directly induce memory effects following light exposure; however, while prolonged charge retention ensures weight retention, it may also lead to baseline drift, difficulty in erasure, and array non-uniformity. Heterojunction and composite channel structures are better suited for extending response to visible or near-infrared light and can enhance light-induced weight changes through interfacial charge transfer; however, interfacial complexity and material stability can affect cycling reliability. While light absorption enhancement in all-oxide composite structures may not be as pronounced as in quantum dot or organic hybrid systems, they are better suited for long-term integration in terms of process compatibility, environmental stability, and array uniformity. Charge-storage and ferroelectric gate-controlled structures are better suited for programmable, multi-level, and long-retention-time synaptic functions, but they face challenges such as polarization fatigue, leakage current, and polarization differences between devices. Therefore, the advantages and disadvantages of specific optoelectronic synaptic device structures should be evaluated in conjunction with the application’s requirements for retention time, erasability, power consumption, cycling stability, and array uniformity.

These characteristics give MO TFT OESs significant application potential in intelligent vision systems. They can directly record optical stimulation history at the perception end and produce time-dependent responses to light intensity, pulse intervals, and continuous stimulation processes. Therefore, their functions can be extended from simple optical signal detection to visual information preprocessing, such as image contrast enhancement, light intensity adaptation, short-term visual memory, motion trajectory perception, and pattern recognition. By combining light perception, state retention, and electrical regulation at the device level, OES devices are expected to reduce data transmission between image acquisition, storage, and computing units, providing a foundation for low-power brain-inspired vision hardware.

Existing research has shown that MO TFT OESs can simulate synaptic weight changes through multi-level conductance modulation and be further used in artificial neural networks and visual recognition tasks [95,116,117,118]. The conductance accumulation induced by light pulses can be used for grayscale adjustment and image enhancement. The slow decay after illumination can simulate visual memory and forgetting processes. Gate voltage or electrical pulses can be used to adjust the initial state and reset process of the device [117,118,119]. These functions enable OES devices not only to perceive external optical signals but also to perform preliminary screening, compression, and storage of input information, approaching the synergistic working mode of perception and processing in biological visual systems.

Overall, OES devices transform the photoresponse process in MO TFTs into a state regulation process with memory and plasticity, providing a new implementation idea for intelligent vision hardware. The next chapter will further discuss the integrated relationship between photodetection and OES functions, focusing on the synergistic implementation methods at the single-device, array, and system levels, as well as their development trends for intelligent vision applications.

## 5. Integration of PDs and OESs with TFTs

The previous two chapters respectively discussed MO TFT PDs and OESs. The former focuses on the detection and conversion of optical signals, while the latter pays more attention to the retention, regulation, and plasticity changes in the conductance state after optical stimulation. Although the two types of devices target different functional goals, their operating processes are closely related to factors such as light absorption, photogenerated carrier transport, defect trapping/release, interface charge modulation, and gate voltage regulation. Therefore, in the MO TFT platform, photodetection and OESs are not completely independent research directions but can form further correlations based on a common photoelectric physical foundation.

With the development of intelligent vision systems, the front end of the device no longer only needs to complete optical signal acquisition but also needs to undertake certain information screening, storage, and preprocessing functions at the perception end. MO TFTs possess the foundation for photoresponse, electrical regulation, and array fabrication, providing possibilities for synergy among photodetection, memory retention, and neuromorphic processing. Based on the research progress of PD and OES devices described above, this chapter further discusses the integration relationship between the two at the array, and system levels, and analyzes their development trends for intelligent vision applications.

### 5.1. Correlation Basis of PD and OES Functions

The discussions in the previous two chapters show that the PD and OES functions in MO TFTs are not independent of each other. Many devices behave as photodetectors when evaluating photoresponse, but when the research focus shifts to the retention, accumulation, and reversible regulation of the conductance state after illumination, they can be further used to simulate OES behavior. The difference between the two does not entirely depend on the device structure itself, but is more reflected in the functional definition and evaluation method of the same photoresponse process.

From existing research, some MO TFTs were initially studied as PDs, but their current retention after illumination, cumulative response to light pulses, and gate-voltage-tunable characteristics provide further opportunities for utilizing synaptic behavior. Conversely, many OES TFTs also rely on basic PD capability as a functional foundation. Only when the device can effectively respond to a specific band or light intensity can subsequent short-term memory, long-term retention, and multi-level weight modulation have a physical premise. Therefore, the detection function can be regarded as the basic link of the OES function, while the synaptic function is a functional extension after introducing a time dimension and state regulation based on the photoresponse [101,116,117,118].

This relationship also indicates that PDs and OESs in MO TFTs do not necessarily correspond to two completely different device designs. By adjusting the channel material, defect state, interface quality, gate dielectric structure, and applied bias, the same type of device can exhibit different emphasis between fast, stable PD and OES behavior with retention characteristics. For applications requiring real-time detection, more attention should be paid to low dark current, high detectivity, and fast recovery. For intelligent vision and neuromorphic computing applications, it is necessary to moderately retain the light-induced conductance state and enable it to be continuously and reversibly regulated.

Thus, PDs and OESs are not isolated research directions. For MO TFTs, the photoresponse can serve both high-sensitivity detection and image acquisition, as well as being transformed into memory retention and state regulation on appropriate time scales. Different studies can focus on one type of function according to application requirements, or further achieve functional synergy at the array or system level.

### 5.2. Perception, Storage, and Visual Preprocessing at the Array Level

After clarifying the relationship between PD and OES functions at the single-device level, array integration is an important step for MO TFTs to further target intelligent vision applications. Visual information typically exists in a spatial distribution form. A single device can only reflect local optical signal changes, while an array structure can convert the photoresponse of different pixel positions into a spatial current distribution, thereby achieving image acquisition, light intensity mapping, and visual information representation. For MO TFTs, their low-temperature fabrication, large-area uniformity, and pixelated processing foundation make them suitable for constructing optoelectronic device arrays for image sensing and front-end processing.

In practical research, MO TFT arrays are often used for PDs and image sensing. Such arrays convert incident light information into analyzable electrical signals through the response differences of different pixel units to light intensity, wavelength, or spatial patterns. For applications such as UV detection, visible light detection, or broadband perception, array performance depends not only on the responsivity and dark current of individual devices but also on pixel-to-pixel consistency, signal stability, and spatial resolution capability. Therefore, stable photoresponse and small device-to-device variation are the foundation for PD arrays to be further used for image acquisition and visual perception [121,122].

For array-based applications, inter-device variability must be considered a key evaluation factor to ensure the results closely reflect actual systems. Even if individual pixels exhibit high responsivity or low dark current, differences in Vth, I_dark_, responsivity, noise levels, and recovery processes among pixels can still lead to fixed-pattern noise, uneven image grayscale, and readout errors [123]. For photonic synapse arrays, inter-device variability also affects the resolvability of multi-level conductance states and the consistency of weight updates, thereby reducing the reliability of subsequent recognition or preprocessing tasks. Therefore, in addition to demonstrating image perception or recognition capabilities, array research should further report inter-pixel variability, yield, long-term drift, and readout stability to ensure that single-device performance can be more reliably translated into array-level application capabilities.

The array readout method also affects the actual performance of optoelectronic TFT devices. Passive cross-array structures are simple, but in large-scale arrays they may be affected by bypass current, crosstalk, and readout errors; active-matrix readout can reduce crosstalk through pixel-level TFT selectors and offers good compatibility with display backplane processes, but it also places higher demands on Vth uniformity, gate/data line driving, and peripheral readout circuits. For OES arrays, if multiple pixels are simultaneously in different holding states, the residual conductance of unselected pixels may further introduce readout background noise, affecting weight mapping and image preprocessing accuracy. Therefore, array design must not only focus on the light response of individual devices but also comprehensively optimize addressing schemes, readout timing, pixel isolation, calibration methods, and peripheral circuit noise.

Park et al. reported an IGZO/SnS_x_/IGZO heterojunction phototransistor array, utilizing the low dark current of IGZO and the light absorption characteristics of SnS_x_ to achieve a stable PD array, providing a relatively new representative example of oxide TFT array-based optical perception [124]. Na et al. further reported a 21 × 7 IGZO phototransistor array, achieving multispectral color perception by introducing a spectral selection structure, demonstrating that oxide phototransistor arrays can be used for wavelength-resolved and high-fidelity image perception [125].

In OES arrays, conductance retention after illumination and history-dependent response provide new ways for visual information processing. Qi et al. reported a room-temperature fabricated a-IGZO OES TFT, which was transformed into pixel weight storage and visual information processing capability at the array level [126]. Li et al. fabricated a large-area ZnO OES TFT array. The devices exhibited good array uniformity, OES behavior, and visual learning and information storage capabilities based on PPC, indicating that pixel-level conductance retention is of great significance for array-based visual memory [127]. Different pixel units can form different conductance states based on the number of light pulses, illumination time, or light intensity differences, thereby preserving certain spatial and temporal information in the array. This characteristic makes OES arrays suitable for front-end processing tasks such as image contrast enhancement, grayscale adjustment, visual adaptation, and short-term memory. Compared with simply increasing the photoresponse amplitude, state retention, response repeatability, and pixel-to-pixel consistency in the array have a more direct impact on visual information processing results [128].

Visual preprocessing is an important direction for MO TFT arrays to further target intelligent vision applications. Traditional vision systems typically need to transmit acquired image data to back-end storage and processing units, while OES arrays can perform part of the information screening and preliminary processing at the perception end. For example, cumulative photoresponse can be used to enhance repeatedly occurring or stronger optical signals, current decay processes can be used to simulate forgetting and temporal filtering, and gate voltage regulation can change the operating state of the array to adapt to different illumination backgrounds. By completing part of the information compression and feature enhancement at the front end, redundant data transmission can be reduced, and the efficiency of subsequent visual recognition and neuromorphic computing can be improved [127,129].

Overall, MO TFT arrays provide a device foundation for intelligent vision systems ranging from optical signal perception to front-end information processing. PD arrays facilitate stable image acquisition and spatial optical signal characterization, while OES arrays can further introduce memory retention, temporal response, and visual preprocessing capabilities. With further optimization of array consistency, pixel size, driving methods, and signal acquisition methods, MO TFTs are expected to play a more important role in image sensing, brain-inspired vision, and edge intelligent perception.

### 5.3. Key Issues and Development Directions in System Integration

After achieving light perception, state retention, and visual preprocessing at the array level, when MO TFTs further target intelligent vision systems, they need to be co-designed with peripheral circuits, signal processing methods, and neuromorphic algorithms. Individual devices or small-scale arrays can verify functions such as PD, synaptic plasticity, and image preprocessing, but system applications are more concerned with whether these functions can operate stably in large-area arrays and form effective connections with back-end computing processes. Therefore, system integration is not simply an increase in the number of devices, but involves matching between device characteristics, array addressing, circuit interfaces, and algorithm mapping.

For PD arrays, the focus of system integration is usually on stably acquiring high-quality image signals and converting pixel currents into data that can be subsequently processed. The responsivity, dark current, noise, and pixel-to-pixel consistency of the device directly affect imaging quality and signal recognition results. For OES arrays, system integration also needs to consider how the conductance state corresponds to weights in neural networks, and how light pulses, voltage pulses, and readout conditions match the training or inference process. If the device conductance update process has strong nonlinearity, drift, or device-to-device variations, the back-end algorithm needs to perform corresponding compensation. Conversely, reasonable algorithm design can also reduce the dependence on the ideality of individual devices [130,131,132].

When applying this approach to actual smart vision hardware, the non-ideal nature of the devices must be considered in conjunction with the circuitry and algorithms. Weight updates in OES devices are often not fully linear, and the enhancement and suppression processes may also exhibit asymmetry, which can cause a deviation in the mapping between conductance states and algorithmic weights. As the array scale increases, device variations, retention drift, and random current fluctuations may further amplify these errors, leading to fluctuations in image preprocessing results or a decrease in recognition accuracy. Lee et al. conducted a quantitative analysis of the impact of weight update asymmetry on the training performance of neural networks, pointing out that update asymmetry can lead to dynamic bias during training and affect the results of weight optimization [133]. In addition, the discrete nature of synaptic device states and inter-device variations can also affect classification results. Kim et al. investigated the effect of synaptic device variability on the recognition accuracy of hardware neural networks [130]; subsequently, Kim et al. further analyzed the impact of synaptic device parameter fluctuations on the classification accuracy of binary neural networks [132]. Therefore, algorithm validation should take actual device characteristics into account as much as possible, rather than relying solely on ideal weight models.

To address these issues, system-level optimization must be advanced through a coordinated approach involving both readout and training methods. At the circuit level, the effects of Vth non-uniformity, I_dark_ drift, and weight decay can be mitigated through pixel calibration, reference cells, differential readout, or periodic refreshing. At the algorithm level, device non-ideal models or noise-aware training can be introduced to enhance the network’s tolerance to limited retention, nonlinear updates, and random fluctuations. For vision front ends operating over the long term, it is also necessary to further evaluate the output stability of the devices under repeated illumination, repeated write–erase cycles, and varying temperature conditions.

From the perspective of process integration, MO TFTs are highly compatible with display backplanes and low-temperature, large-area fabrication processes, which lays the foundation for building large-area visual sensing arrays. However, to further integrate with CMOS readout circuits or display driver systems, attention must still be paid to issues such as pixel current range, readout noise, addressing methods, packaging stability, and process thermal budget. Therefore, future system integration research should gradually shift from demonstrating single-device functionality to the joint validation of devices, arrays, readout circuits, and algorithmic tasks.

In intelligent vision systems, MO TFT devices can undertake functions at different levels. Some systems focus more on front-end optical signal acquisition, obtaining stable image input through high-sensitivity PD arrays. Others emphasize perception-end preprocessing, using OES arrays to achieve image enhancement, temporal filtering, visual adaptation, or simple classification. Still, other designs may distribute detection, storage, and computing units, achieving a more complete information processing chain through circuit connections.

Lee et al. pointed out in a review that OES devices can complete image preprocessing at the sensing end, such as contrast enhancement, image filtering, and feature extraction, thereby reducing back-end data transmission and computational pressure [129]. Meng et al. proposed an integrated in-sensor computing optoelectronic device, achieving light adaptation, image preprocessing, and handwritten digit recognition, reflecting the application potential of integrated perception, storage, and processing devices in artificial retina systems [134]. Liao et al. further achieved visual adaptation through phototransistors with time-dependent activation and inhibition characteristics, demonstrating that front-end device response characteristics can be combined with visual algorithm tasks to improve perception accuracy under different illumination conditions [135].

Overall, MO TFTs provide a material and device foundation for intelligent vision systems ranging from optical signal perception to front-end processing. The development of their system integration should not solely target individual performance metrics, but should pay more attention to the coordination among device function, array consistency, circuit interface, and algorithm requirements. With the advancement of large-area fabrication, low-power driving, and device–circuit–algorithm co-design, MO TFTs are expected to play a more important role in image sensing, visual preprocessing, edge intelligence, and brain-inspired vision systems.

## 6. Summary and Outlook

The preceding sections discussed the physical basis, device implementation, and intelligent vision applications of MO TFT PDs and OESs. It can be seen that the photoelectric behavior of MO TFTs serves not only optical signal detection but can also be further used for state retention and synaptic regulation. This chapter summarizes the entire text and provides an outlook on key directions that still require attention in this field.

### 6.1. Summary

This review discussed MO TFT PDs and OESs. Metal oxide semiconductors have characteristics such as wide bandgap, good transparency, low-temperature processing, and large-area process compatibility, making them valuable for research in TFT optoelectronic devices. In the TFT structure, gate regulation can change the initial channel state and interface electric field, so that photogenerated carrier transport, defect trapping/release, and interface charge modulation processes are no longer determined solely by the material itself but can act together with the applied electric field. This three-terminal regulation characteristic gives MO TFTs more flexible operating modes in PD and OES applications, and also provides additional means for optimizing device performance and expanding functionality in the future.

In terms of PD, research on MO TFTs has gradually expanded from intrinsic UV response to DUV, near-UV, visible light, near-infrared, and broadband detection. Wide-bandgap and ultra-wide-bandgap oxides are suitable for DUV and SBUV detection, while near-UV and UV detection pay more attention to response stability and defect relaxation processes. For visible and near-infrared detection, the intrinsic absorption of single wide-bandgap oxides is limited, typically requiring defect-state engineering and composite structure design to extend the low-energy photoresponse. As detection requirements evolve from single-band to broadband and multi-band identification, device design has gradually shifted from simply increasing response amplitude to balancing spectral selectivity, cross-band stability, and signal distinguishability.

In terms of OESs, MO TFTs extend the photoresponse from instantaneous current changes to the temporal evolution of the conductance state. By adjusting defect states, interface charges, and applied bias, the conductance retention and relaxation processes after illumination can be used to simulate synaptic weight updates and memory behavior. The core of related research lies in controlling the generation, retention, and recovery processes of the light-induced conductance state. Therefore, channel defects, interface trapping, charge storage, ferroelectric gate control, and composite channel design have all become important approaches for regulating synaptic behavior.

The PD and OES functions reflect the functional differentiation of the photoresponse process in MO TFTs under different application goals. Many devices can behave as PDs under different evaluation targets and can also be further used to simulate OES behavior. The detection function provides the foundation for optical signal acquisition, while the synaptic function introduces a time dimension and state regulation based on the photoresponse. For intelligent vision applications, MO TFT arrays can further undertake perception-end information processing functions on the basis of optical signal acquisition, and through combination with peripheral circuits and neuromorphic algorithms, promote the development of low-power vision systems.

MO TFTs provide a research platform for PD and OES functions that combines material tunability, structural designability, and process compatibility. Current research has made significant progress in multi-band photoresponse, synaptic plasticity simulation, and intelligent vision function verification. Subsequent development still needs to continuously advance around directions such as controllable regulation of material defects, interface and gate dielectric optimization, composite structure stability, array consistency, and device–circuit–algorithm co-design, to promote MO TFT optoelectronic devices from single-device function demonstration to more reliable integrated intelligent vision applications.

### 6.2. Outlook

MO TFT PDs and OESs have made considerable progress in multi-band photoresponse, synaptic function regulation, and intelligent vision function validation. However, their further development still needs to be promoted synergistically from multiple levels: material, device, array, and system.

First, the directional design of materials and defects remains the foundation for improving device performance. Oxygen vacancies, band tail states, deep-level defects, and interface states in metal oxide semiconductors affect not only the dark current, response speed, and stability of PDs but also determine the conductance retention and weight update processes in OES devices. Subsequent research needs to move from empirical process regulation to controllable defect design, combining spectroscopic characterization, electrical analysis, and physical modeling to clarify the roles of different defect states in photoresponse enhancement, PPC, and conductance recovery. To address the insufficient response to visible and near-infrared light, it is also necessary to improve low-energy light absorption efficiency and carrier separation efficiency through material composition regulation, composite channels, and heterointerface design. Ga_2_O_3_ TFTs have been used to combine ultraviolet sensing with information processing [36]. Therefore, the optimization of materials and defects should be based on setting defect states according to detection, memory, or computational tasks, rather than focusing solely on reducing defect concentration or improving responsivity as evaluation criteria.

Second, device structure and multi-function synergy still have room for further optimization. The gate electrode in the TFT structure provides an important means for photoresponse regulation. Subsequent designs can use high-k gate dielectrics, dual-gate structures, charge storage layers, ferroelectric gate dielectrics, and composite channels to modulate channel potential, interface charges, and conductance retention processes. Different applications impose different requirements on device structure. PDs focus more on stable conversion and fast recovery of photoresponse, while OESs emphasize controllable retention and reversible update of the conductance state.

Third, array stability and engineering compatibility are important prerequisites for MO TFTs to move towards intelligent vision applications. Excellent single-device performance does not necessarily imply stable array performance. Pixel-to-pixel variations, process fluctuations, environmental adsorption, and long-term bias stress can all affect the results of image acquisition, visual preprocessing, and neuromorphic computing. Therefore, subsequent research needs to pay more attention to device consistency, repeatability, and long-term reliability under large-area fabrication conditions, and establish testing methods suitable for array evaluation. Based on the current state of the applications, oxide TFTs have already established a relatively mature technological foundation in the field of display backplanes. Research on large-area LCD displays and AMOLED backplanes using IGZO-TFTs has shown that these devices can meet the requirements of high-resolution displays for large-area uniformity, low leakage current, and stable pixel driving [136,137]. This application experience demonstrates that metal-oxide TFTs already possess the foundation for large-scale array manufacturing and circuit integration, providing the practical conditions for their further expansion into optoelectronic detection arrays and smart vision front ends. The combination of low-temperature processes, flexible substrate compatibility, and existing display or sensing process technologies will also affect the practical application potential of MO TFT optoelectronic devices.

Furthermore, device–circuit–algorithm co-design will become a key direction for promoting development in this field. The output of MO TFT optoelectronic devices typically has nonlinearity, history dependence, and certain device-to-device variations. These characteristics can both introduce systematic errors and be reasonably utilized for visual preprocessing and neuromorphic computing. In the future, a closer correspondence needs to be established between device characteristics and circuit architecture, signal readout methods, and algorithm models, so that the device’s photoresponse, conductance retention, and weight update processes can better serve practical computing tasks. Through algorithm compensation, array calibration, and hardware-friendly training methods, the dependence on the ideality of individual devices can be reduced, and overall system performance can be improved.

Finally, standardized evaluation and application scenario expansion also need to be further strengthened. Currently, there are still large differences in illumination conditions, bias settings, device dimensions, energy consumption calculations, stability testing, and synaptic performance evaluation across different studies, making direct performance comparisons between different devices less straightforward. Pecunia et al. pointed out in a consensus article on the accurate evaluation of PDs based on emerging semiconductors that PD performance characterization requires clarifying test conditions, correctly calculating key indicators, and avoiding parameter misuse [138]. On this basis, MO TFT-based optoelectronic devices also need to further improve evaluation methods for OES and array applications, enabling photoresponse capability, state retention behavior, and array reliability to be analyzed under more comparable conditions.

In summary, MO TFTs provide a tunable and engineering-compatible device platform for PD and OES functions. The future development of this field requires the formation of closer links among material defect control, device structure design, array reliability, and system synergy, thereby promoting MO TFT-based optoelectronic devices from single-device function verification to stable, low-power, and integrable intelligent vision applications.

## Figures and Tables

**Table 1:** Summary of key parameters for metal oxide TFT photodetectors.

Materials	Preparation	Response Range (nm)	R (A/W)	PDCR	t_rise_	t_fall_	Ref.
β-Ga_2_O_3_	MOCVD	254	20.73	2.2 × 10^5^	/	0.3 s	[36]
a-Ga_2_O_3_	Magnetron Sputtering	254	8.6	~10^3^	/	/	[38]
κ-Ga_2_O_3_	PLD	250	703	1.66 × 10^7^	0.81 s	0.14 s	[39]
ε-Ga_2_O_3_	MOCVD	254	6.18	1.45 × 10^5^	0.14 s	0.09 s	[40]
a-Ga_2_O_3_:CdO	Solution Method	260	2.17	1.6 × 10^3^	/	/	[41]
a-Ga_2_O_3_	Magnetron Sputtering	260	13	/	/	/	[42]
a-Ga_2_O_3_	Magnetron Sputtering	254	832.8	6.01 × 10^5^	2.1 s	9.8 s	[43]
a-Ga_2_O_3_	Magnetron Sputtering	254	7.8 × 10^5^	~10^10^	0.48 s	1.97 s	[45]
a-Ga_2_O_3_	Magnetron Sputtering	254	5.77 × 10^5^	4.93 × 10^7^	3.59 s	7.54 s	[46]
MgZnO	Magnetron Sputtering	290	3.12	4.4 × 10^5^	/	/	[49]
ZnGaO	PEALD	270	34.31	2.2 × 10^7^	6.64 s	0.01 s	[52]
a-IGZO	Magnetron Sputtering	250	6.93	3.7 × 10^4^	/	/	[54]
ZnO	Hydrothermal Method	365	17.4	224.8	6.2 s	240 s	[55]
In_2_O_3_/PbI_2_	Solution Method	395, 445	/	750@395 nm 152@445 nm	3.7 s	24 s	[56]
PEDOT:PSS/SnOx/IGZO	Magnetron Sputtering, Spin Coating	320	984	10^3^	/	/	[64]
ZnSnON	Magnetron Sputtering	200–800	6 × 10^3^ (400–800 nm) ~10^5^ (UV)	/	/	/	[66]
ZnSnON	Magnetron Sputtering	350–650	1097@450 nm, 868@550 nm, 253@650 nm	/	0.38 s	0.53 s	[67]
a-IGZO	Magnetron Sputtering	white LED	0.95 mA/W	~8000	1.2 s	18 s	[68]
a-IGZO/ITON	Magnetron Sputtering	450–635	4.74 × 10^4^@450 nm, 4.06 × 10^3^@532 nm, 30.2@635 nm	1.49 × 10^8^@450 nm, 1.03 × 10^7^@532 nm, 6.34 × 10^4^@635 nm	/	/	[76]
a-IGZO/C8-BTBT	Magnetron Sputtering, Spin Coating	360–1200	1.21@360 nm	/	8 ms	12 ms	[84]
IZO/BHJ	Inkjet Printing, Spin Coating	500–1400	/	/	~ms	/	[86]

**Table 2:** Summary of key parameters for metal oxide TFT optoelectronic synapse.

Materials	EPSC	PPF Index	Retention Time	Endurance	Linearity	Number of Conductance States	Ref.
IGCO	~7 nA	7.49/79.85	56.8 s	/	/	/	[9]
IGZO	/	35.9/35.5	12.5 s (90 pulses)	/	/	90	[103]
TiO_2_	/	/	/	/	/	128	[106]
TiO_2_	~10^−7^ A	/	32.3 s (0.5 s pulse)	/	0.9	1024	[107]
IGZO	/	/	/	/	0.27	2000	[108]
IGZO	35.67 μA	137%	500 s	>100 cycle	0.25 (Potentiation) 1.6 (Depression)	12.13	[109]
IGZO	14 nA	208%	26.2 s	>10 cycle	0.56 (LTP); 2.08 (LTD)	8	[112]
IGZO	35.7 nA	/	200 s	>8 cycle	charge trapping: 0.47 (LTP); 0.39 (LTD) ferroelectric polarization: 4.24 (LTP); 4.53 (LTD)	>10	[113]
IGZO	3.5 µA	/	50 s	10,000 cycle	4.81 (LTP)	7.54 s	[117]
InWZnO	~23.4 nA	176%	160 s	/	0.102 (LTP); 0.32 (LTD)	4	[95]

## Data Availability

No new data were created or analyzed in this study. Data sharing is not applicable to this article.
